# Large-scale conformational changes and redistribution of surface negative charge upon sugar binding dictate the fidelity of phosphorylation in *Vibrio cholerae* fructokinase

**DOI:** 10.1038/s41598-018-35236-3

**Published:** 2018-11-16

**Authors:** Rakhi Paul, Shramana Chatterjee, Seema Nath, Udayaditya Sen

**Affiliations:** Crystallography and Molecular Biology Division, Saha Institute of Nuclear Physics, HBNI, 1/AF Bidhan Nagar, Kolkata, 700064 India

## Abstract

Fructokinase (FRK) catalyzes the first step of fructose metabolism i.e., D-fructose to D-fructose-6-phosphate (F6P), however, the mechanistic insights of this reaction are elusive yet. Here we demonstrate that the putative *Vibrio cholerae* fructokinase (*Vc*FRK) exhibit strong fructose-6-kinase activity allosterically modulated by K^+^/Cs^+^. We have determined the crystal structures of apo*-Vc*FRK and its complex with fructose, fructose-ADP-Ca^2+^, fructose-ADP-Ca^2+^-BeF_3_^−^. Collectively, we propose the catalytic mechanism and allosteric activation of *Vc*FRK in atomistic details explaining why K^+^/Cs^+^ are better activator than Na^+^. Structural results suggest that apo *Vc*FRK allows entry of fructose in the active site, sequester it through several conserved H-bonds and attains a closed form through large scale conformational changes. A double mutant (H108C/T261C-*Vc*FRK), that arrests the closed form but unable to reopen for F6P release, is catalytically impotent highlighting the essentiality of this conformational change. Negative charge accumulation around ATP upon fructose binding, is presumed to redirect the γ-phosphate towards fructose for efficient phosphotransfer. Reduced phosphotransfer rate of the mutants E205Q and E110Q supports this view. Atomic resolution structure of *Vc*FRK-fructose-ADP-Ca^2+^-BeF_3_^−^, reported first time for any sugar kinase, suggests that BeF_3_^−^ moiety alongwith R176, Ca^2+^ and ‘anion hole’ limit the conformational space for γ-phosphate favoring in-line phospho-transfer.

## Introduction

Phosphorylation of monosaccharides is a fundamental reaction in carbohydrate metabolism which traps sugar inside the cells and targets them for further utilization by specific metabolic pathways. Although the main pathway of sugar phosphorylation in bacteria involves classic phosphotransferase system (PTS) dependent enzymes^1^, a PTS-independent nucleotide (NT) dependent pathway is also used by several bacteria. NT dependent sugar kinases are divided into three distinct non-homologous families: hexokinase (HK), ribokinase and galactokinase^2^. Later, a fourth family, namely ROK (Repressor, ORF, Kinase) sugar kinases was identified^3^. Fructose, a highly abundant monosaccharide in nature, not only used as a major energy source but also serves as a vital carbon source in bacteria. Although fructose can be phosphorylated by HK similarly to glucose, the relative affinity of HK for fructose is substantially low and under most conditions fructose is phosphorylated by fructokinase, an enzyme specific for fructose^2^. Fructokinases can be categorized broadly into three distinct classes having widely different in structure and mode of phosphorylation. In mammals, hepatic fructokinase (EC 2.7.1.3) phosphorylate fructose to fructose-1-phosphate (F1P) and initiates fructose catabolism through a specialized pathway that bypass the major glycolytic checkpoint at phosphofructokinase^4^. ROK fructokinase, on the other hand, phosphorylates fructose to fructose-6-phosphate (F6P). ROK family of sugar kinases are widespread in nature and found almost in all species from bacteria to humans^3^. Another fructokinase, FRK (EC 2.7.1.4), belongs to pfkB family of sugar kinase, catalyzes the phosphorylation of D-fructose to D-fructose-6-phosphate (F6P). This irreversible and near rate-limiting step is a central and regulatory process in plants and bacteria, which channels fructose into a metabolically active form for glycolysis^4^. The β-D form of F6P lies within the glycolytic pathway and is very common in cells. Vast majority of glucose and fructose entering a cell becomes converted to this form at some point^4^.

Structural perspective of catalytic phosphotransfer has been enlivened for several pfkB family of enzymes which include RK^5^, Adenosine kinase (AK)^6^, 4-methyl-5-beta-hydroxyethylthiazole kinase (ThiK)^7^, 4-amino-5-hydroxymethyl-2-methylpyrimidine phosphate kinase (HMPPK)^8^ and Aminoimidazole Riboside kinase (AIRsK)^9^. For the phosphotransfer reaction both divalent and monovalent cations, Mg^2+^ and K^+^ physiologically, are required for catalysis and allosteric activation^7–10^. However, detailed understanding about the precise positioning of the γ-phosphate during phosphotransfer or the atomistic details of the allosteric activation mechanism is insufficient to date. Among the fructokinases structure and mechanistic details of hepatic fructokinase^11^ and ROK fructokinase^12^ are known, but no information is available yet to understand the mechanisms underlying fructose phosphorylation by FRK. Only structural information about FRK family is the crystal structure of *Halothermothrix orenii* in apo form (2.8 Å) where several important catalytic loops were poorly defined^13^. Therefore, thorough investigations are required to delineate the mechanistic details of fructose phosphorylation by FRK.

The facultative human pathogen *Vibrio cholerae* adapts to various hostile situations by the timely coordinated expressions of genes. High growth rate of *V*. *cholerae* after colonization in human host requires highly active metabolic machinery. At the late stages of infection, on the other hand, induction of a fructose-6-phosphate uptake system is required that build up endogenous phosphate storage for better fitness to phosphate-limiting environments^14^. *V*. *cholerae* FRK (*Vc*FRK), encoded by *cscK* gene was predicted to have fructokinase activity according to KEGG pathway. Our isothermal titration calorimetry (ITC) data demonstrated that *Vc*FRK specifically binds D-fructose while kinetic experiments confirmed strong divalent metal dependent fructokinase activity where monovalent cations as allosteric activators. We have also determined the crystal structures of *Vc*FRK in apo form and in complex with fructose, fructose-ADP-Ca^2+^, fructose-ADP-Ca^2+^-BeF_3_^−^. Collectively, we propose a mechanism of fructose phosphorylation by *Vc*FRK and its allosteric activation. Large scale (>20 Å) conformational changes of *Vc*FRK have been envisaged to sequester and trap the fructose which needs to bring back during F6P release. Structural and kinetic studies of a double mutant (H108C/T261C-*Vc*FRK), designed to remain tethered in closed form upon fructose binding, is seen catalytically impotent implying this conformational changes are essential for catalysis. Binding of fructose triggers an accumulation of negative charges around ATP which we propose bucks-up the γ-phosphate towards fructose and dictates the phosphotransfer fidelity. This idea was verified through several *Vc*FRK mutants and database analysis. BeF_3_^−^ bound structure of *Vc*FRK-fructose-ADP-Ca^2+^ (1.3 Å), reported for the first time for any sugar kinase, illustrates that tetrahedral BeF_3_^−^ moiety is positioned in between β-phosphate of ADP and O6′ of fructose. Interaction of BeF_3_^−^ with Ca^2+^, anion hole and R176 highlights the interaction made by the γ-phosphate during the transition state of phosphotransfer. These interactions, along with several small changes around, would limit the conformational freedom of β/γ phosphates providing a platform for better phosphotransfer.

## Results

### Overall fold of *Vc*FRK monomer

**Figure 1:**
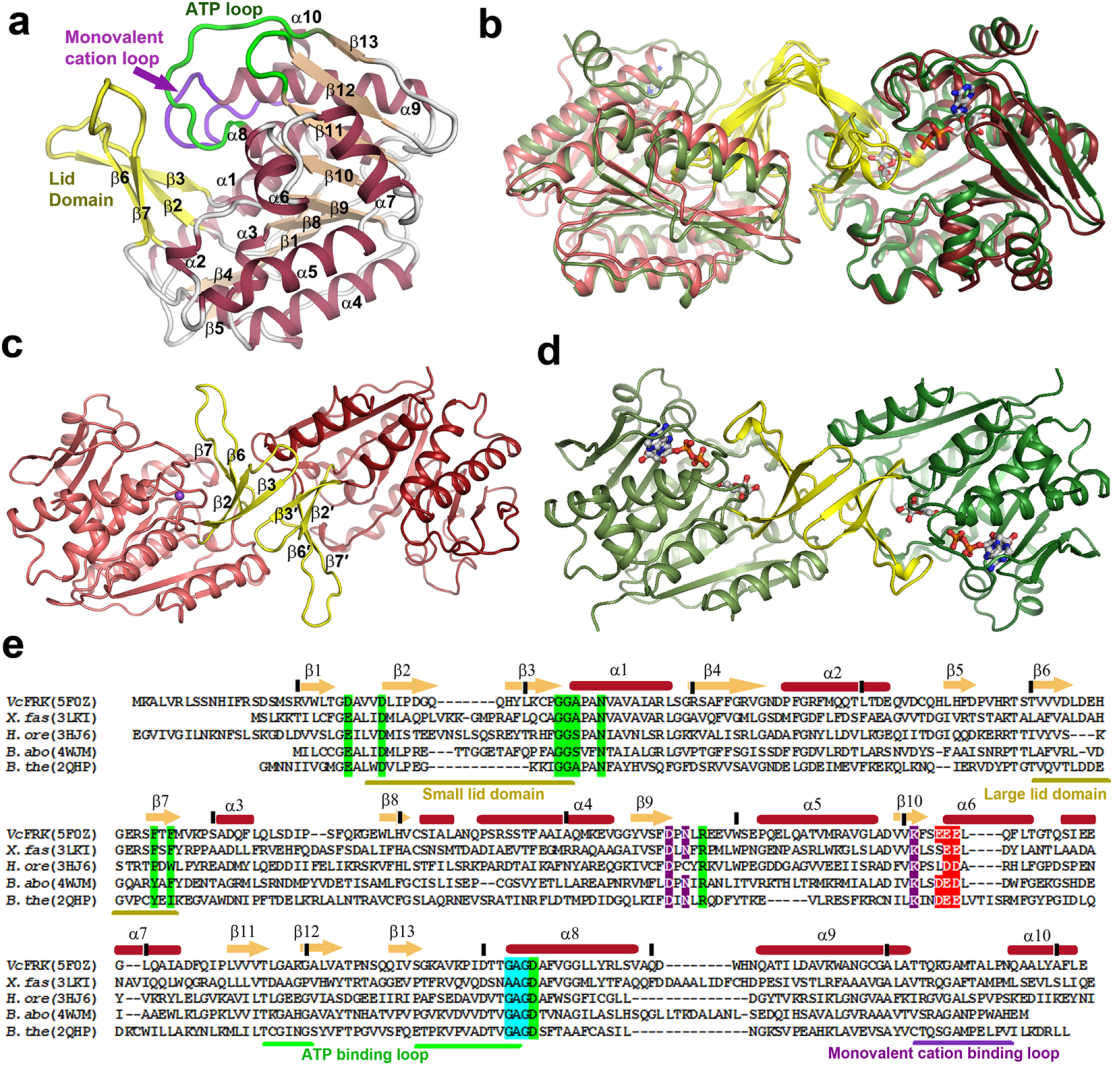
Structure of *Vc*FRK and its dimerization. (**a**) Cartoon representation of *Vc*FRK monomer, ‘Lid’ domains (yellow), important loops involved in phosphorylation, helices (cadbury) and central β-sheet (sand) are labeled. (**b**) Superposition of apo (cadbury shades) and fructose-ADP bound (green shades) dimers of *Vc*FRK in cartoon representation. (**c**) Involvement of lid domains in dimerization of apo and (**d**) fructose+ADP (in sticks) bound *Vc*FRK. Shift of large lid loop upon sugar binding is evident. (**e**) Sequence alignment of *Vc*FRK with other fructokinases overlaid with secondary structures (top). Important loops are indicated (bottom) with same color scheme as (a). Conserved residues involved in sugar binding (green), anion hole formation (cyan), divalent cation binding (violet) and negatively charged patch formation near ATP (red) are indicated.

All the *Vc*FRK structures (apo and ligand bound) were refined well with excellent stereochemical parameters consistent with the quality of the diffraction data (Table 1). Each monomer of *Vc*FRK exhibits the typical ribokinase like fold consisting of a αβα three-layer sandwich domain and a lid domain. The αβα domain contains a central nine-stranded β-sheet, formed by both parallel (β5, β4, β1, β8, β9, β10, β11) and antiparallel (β12, β13) β-strands, surrounded by ten α-helices and two 3_10_ helices (Fig. 1a). One face of the β-sheet is flanked by α4, α5, α6, α7 and two short 3_10_ helices H1 and H2, while the other face, that harbors most of the active site residues, contains rest of the helices. The flexible lid domain, which extends from the central β-sheet core, is formed by two pairs of antiparallel β-strands β2-β3 and β6-β7 and their connecting loops. They are designated as small lid sub-domain and large lid sub-domain respectively. The active site is located at the interface between the lid domain and the αβα domain while the ATP binding site resides between two loops connecting β13 and α8 and β11 and β12 (Fig. 1a).

**Table 1:** Data Collection and Refinement Table.

	*Vc*FRK apo	*Vc*FRK + FRU	*Vc*FRK + ADP + FRU + Ca^2+^ + Na^+^	*Vc*FRK+ADP+FRU + Ca^2+^+Na^+^ + BeF_3_^−^	*Vc*FRK-DM+ADP + FRU + Ca^2+^ + K^+^
**Data collection**					
Wavelength (Å)	1.54	1.54	1.54	0.97	1.54
Space group	P22_1_2_1_	P22_1_2_1_	P22_1_2_1_	P22_1_2_1_	P22_1_2_1_
Unit cell parameters (a/b/c (Å))	107.12/99.86/61.55	117.24/64.67/41.89	107.00/64.29/41.76	64.30/107.22/41.62	41.76/64.33/116.40
Resolution range (Å)*	29.41–2.46 (2.5–2.45)	25.66–2.30 (2.44–2.4)	35.00–1.75 (1.76–1.73)	32.15–1.29 (1.31–1.29)	33.92–1.67 (1.71–1.67)
No. of reflections	23435	15710	29487	71986	36538
R_sym_ (%)	4.59 (21.4)	10.5 (20.2)	5.46 (26.38)	4.4 (46)	5.5(37.8)
I/σ (I)	7.0 (1.8)	7.1 (1.7)	6.7 (1.6)	31.5 (1.7)	9.6 (1.74)
Completeness (%)	92.5 (94.3)	94.0 (96.6)	95.1 (92.2)	97.4 (81.1)	94.7 (87.0)
Redundancy	2.73 (2.76)	3.3 (3.13)	3.15 (3.08)	3.9 (3.4)	3.5 (3.3)
Wilson B-factor (Å^2^)	41.7	27.8	16.8	15.7	12.6
**Refinement**					
Rwork/Rfree (%)	21.6/24.2	21.1/26.3	14.5/17.9	13.5/15.8	16.31/19.47
No. of atoms (protein/ligand/solvent)	4545/1/83	4622/13/210	2349/41/356	2326 /45/379	2340/41/342
Mean B value (Å^2^)	69.0	34.0	17.0	16.0	18.0
RMS deviation (bond(Å)/angle (deg)/chiral volume (Å)	0.004/0.968/0.029	0.003/0.806/0.077	0.010/1.377/0.077	0.02/1.46/0.069	0.012/1.115/0.067
Ramachandran plot (favored/allowed (%))	90.7/6.1	96.1/2.9	98.7/1.3	98.7/1.3	98.38/1.30
PDB Code	5EY7	5F11	5F0Z	5EYN	5YGG

*Values within parenthesis refer to the highest resolution shell.

### *Vc*FRK dimerize through their lid domain

Crystal structure of *Vc*FRK indicated a dimeric architecture (Fig. 1b–d) in both apo and sugar bound forms. However, unlike apo and sugar bound *Ec*RK, both the forms of *Vc*FRK have comparable dimensions (Fig. 1b). At the centre of the dimeric interface of apo *Vc*FRK, small lid sub-domain from each monomer interact strongly while their large lid sub-domains are projected away, forming eight stranded β-sheet arrangement β7-β6-β2-β3-β3′-β2′-β6′-β7′ (Fig. 1c). Although both *Vc*FRK and *Ec*RK^5^ dimerize through lid domains their mode of dimerization is quite different. While in *Ec*RK lid domains form a β-clasp type of structure in *Vc*FRK they are from a side-by-side dimer or flap kind of structure.

Dimerization of *Vc*FRK buries about 7% of the total surface area (1900 Å^2^ out of total 25510 Å^2^). *Vc*FRK dimer is mostly stabilized through hydrophobic interactions involving residues L31, Y39, P71, F72, V102, M116 and V117 from each monomer and they are nearly conserved in other FRKs (Fig. 1e). In addition several main-chain hydrogen bonds between their β3 strands also stabilize the dimer. Because of two-fold symmetry, these interactions are occurring twice providing a net free energy of interaction −24 kcal/mole as calculated by *PDBePISA* webserver^15^. Dimeric form of *Vc*FRK is also evident in solution (Fig. S1).

### Fructose binding triggers large-scale movement of the lid domain

**Figure 2:**
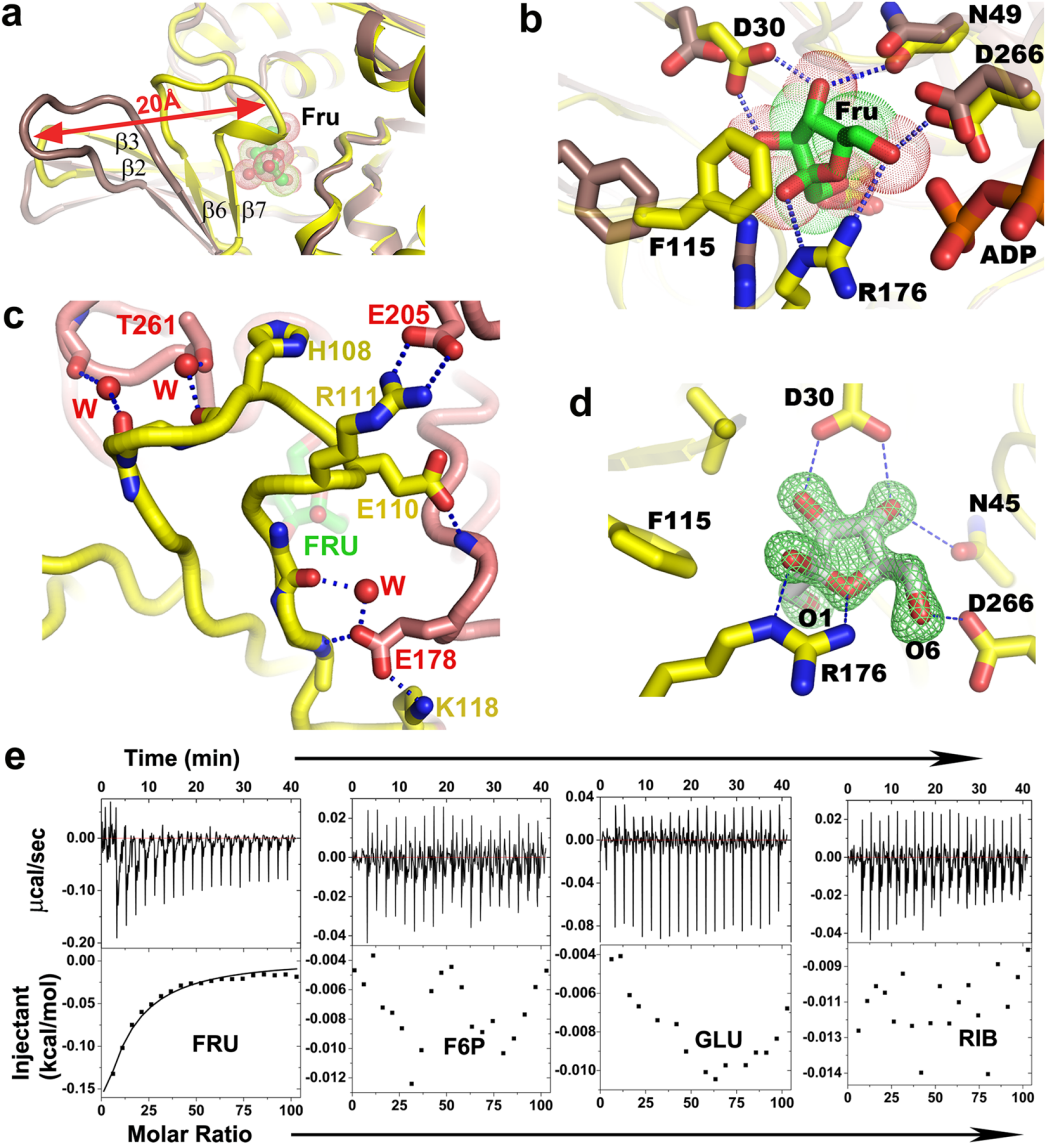
Fructose binding and specificity of *Vc*FRK. (**a**) Movement of the large lid loop upon fructose (green sticks) binding (apo:cadbury, fructose bound:yellow) is shown in red arrow. (**b**) *Vc*FRK residues involved in sequestering fructose through H-bonds. Position of these residues in apo form is also indicated (Cadbury). (**c**) Polar interactions that tether the large lid loop with the αβα sandwich domain. Important residues and water molecules bridging large lid loop and αβα domain are labeled. T261 and H108 are indicated. (**d**) *F*_*o*_ − *F*_*c*_ omit electron density map, contoured at 4σ (green mesh), around fructose(grey sticks). (**e**) Representative ITC titration curve of *Vc*FRK with fructose (FRU), Fructose-6-phosphate (F6P), Glucose (GLU) and Ribose (RIB).

Fructose binds on the top of the highly conserved GGA motif, located at the junction of the lid domain and the central αβα domain. Most striking structural changes occur upon fructose binding where large lid sub-domain folds back about 20 Å towards the αβα domain (Fig. 2a). This structural change facilitates sequestration of fructose by forming extensive H-bonds between D26, D30, N49, R176, D266 and the main-chain N-atoms of G45 and A46 with fructose (Fig. 2b). In addition, F113 and F115 from large lid sub-domain completely bury the fructose through hydrophobic shielding and expose only its O6′ atom poised to attack the γ-phosphate of ATP. Folded large lid sub-domain makes several polar interactions with the αβα domain and traps the fructose (Fig. 2c). Fructose binds in a relatively uncommon C4′-endo configuration of the β-D-furanose form (Fig. 2d) which is only ~22% of the total anomeric form present in solution^16^. Residues involved in fructose binding are stubbornly conserved throughout the FRK family which indicates that sugar-coordinating residues are critical determinant to bind this specific stereoisomer (Fig. 1e). Binding of fructose shapes up the ATP binding site further through several small induced fit movements. However, it has little effect on the overall dimension of *Vc*FRK dimer (Fig. 1b). This is in contrast to *Ec*RK, where an open apo *Ec*RK dimer adopts a closed form upon sugar binding with wide changes in overall dimensions^17^. Both sugar bound/unbound form of *Vc*FRK resembles closed form of *Ec*RK.

### Ligand screening by ITC revealed specificity of *Vc*FRK towards fructose

The affinity of *Vc*FRK towards different sugars is characterized by ITC. Results of ITC revealed the binding affinity of *Vc*FRK for fructose at 1.37 mM with favourable values of enthalpy and entropy changes (Fig. 2e). As expected, F6P exhibits negligible binding affinity. No affinity toward glucose, a hexose sugar with pyranose ring or ribose, a pentose sugar with furanose ring, demonstrating its high level of specificity for the sugars to be phosphorylated.

### Fructokinase activity of *Vc*FRK is influenced by mono/di valent cations

**Figure 3:**
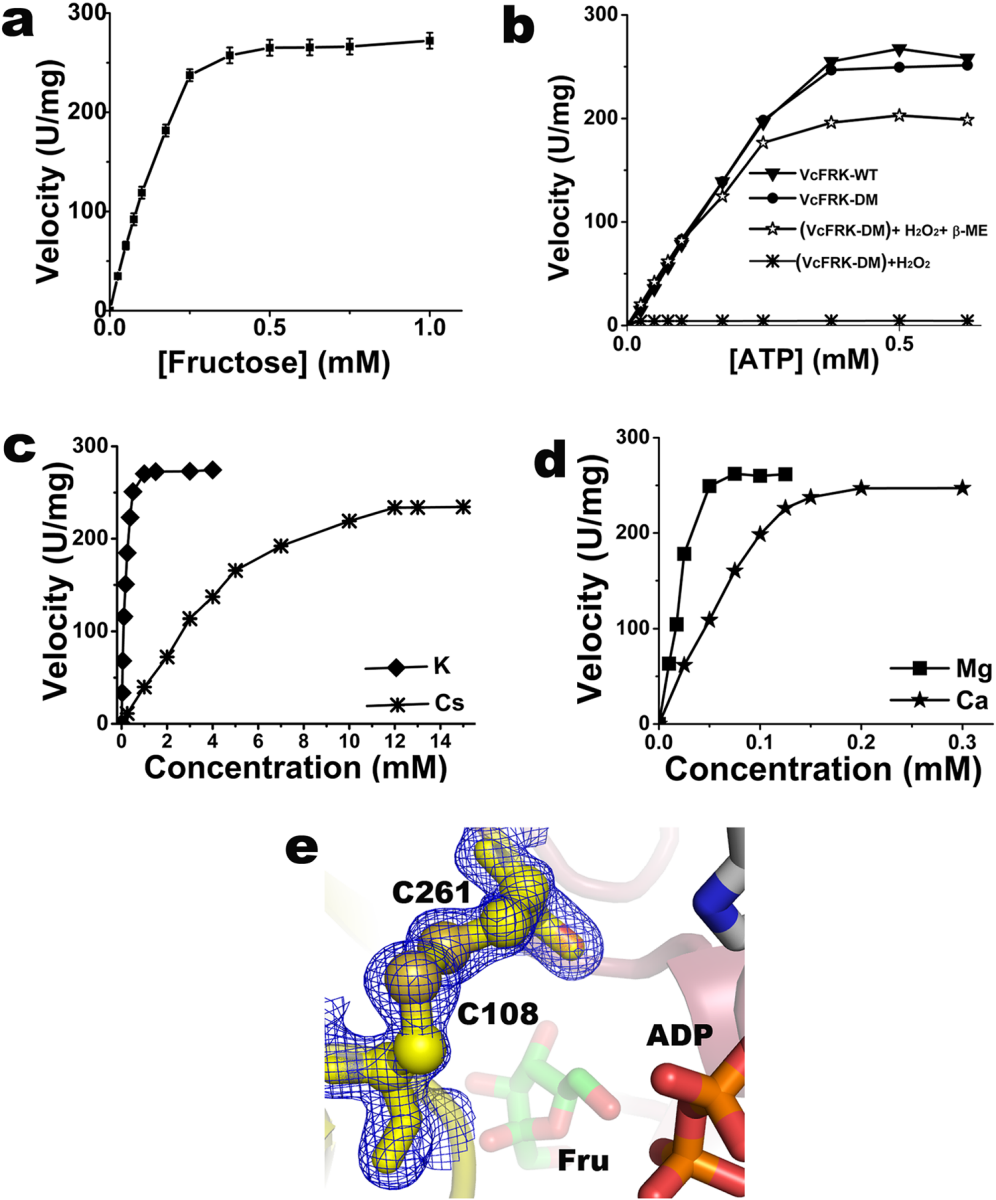
Enzymatic activity of *Vc*FRK. Saturation curve was fit with the Michaelis–Menten equation to obtain estimates of K_m_. Error bars correspond to the standard deviation of three independent measurements. Variation of enzymatic activity of *Vc*FRK with (**a**) fructose, (**b**) ATP (WT and *Vc*FRK-DM in various forms as indicated), (**c**) monovalent cations K^+^ and Cs^+^ and (**d**) divalent cations Mg^2+^ and Ca^2+^, (**e**) Crystal structure of *Vc*FRK showing a disulphide bond between C108 and C261; fructose and part of ADP is also shown. *2F*_*o*_ − *F*_*c*_ electron density map (blue mesh; 1σ) around the disulphide bond is shown.

*In vitro* enzymatic activity of *Vc*FRK was tested under standard reaction conditions. The amount of F6P generated by *Vc*FRK is estimated by coupled phosphoglucose isomerase and glucose 6-phosphate dehydrogenase reactions, and the decrease of NADH was examined spectrophotometrically at 340 nm^18^. *Vc*FRK catalyzes D-fructose to D-fructose-6P with apparent K_m_ and V_max_ values of 0.37 ± 0.02 mM (with respect to fructose) and 275 ± 12 U/mg respectively (Fig. 3a) while the K_m_ for ATP was observed to be 0.28 ± 0.02 mM (Fig. 3b). High turnover number and low K_m_ value indicate that *Vc*FRK is a potent fructokinase. Monovalent cations strongly influences the activation of *Vc*FRK with K_m_ of 0.17 ± 0.01 mM for K^+^ and K_m_ of 9.54 ± 0.07 mM for Cs^+^ (Fig. 3c), but Na^+^ has no detectable activation up to 200 mM NaCl. Divalent cations Mg^2+^ and Ca^2+^ also influence the phosphotransfer with K_m_ values of 0.027 ± 0.001 mM and 0.12 ± 0.01 mM respectively (Fig. 3d).

### Consistent closure/opening of the large lid domain is essential for fructokinase activity

Upon fructose binding large lid domain of *Vc*FRK moves towards αβα domain and traps the sugar. Interactions between the large lid domain and αβα domain should be sufficient to keep the fructose in trapped form as long as it is not phosphorylated but not too strong to hinder the release of the product F6P. Crystal structure of *Vc*FRK-fructose shows that H108 of the large lid domain and T261 of the αβα domain are very close, which were otherwise about 20 Å apart in the apo structure (Fig. 2a,c). To check the essentiality of loop closure and opening, in fructose binding and F6P release respectively, a double mutant H108C/T261C-*Vc*FRK (henceforth designated as “*Vc*FRK-DM”) was designed. Fructose bound *Vc*FRK-DM structure (1.67 Å) shows that C108 and C261 are disulphide linked (Fig. 3e). Hence, *Vc*FRK-DM is expected to remain in closed form upon fructose binding and thus unable to re-open during F6P release. Under reducing condition, *Vc*FRK-DM exhibit comparable kinase activity compared to that of wild type *Vc*FRK (Fig. 3b) implying that H108C/T261C mutations do not hamper the kinase activity. The O6′ atom of fructose that would be phosphorylated remains accessible in both the *Vc*FRK-DM-fructose and *Vc*FRK-fructose structures. However, under mild oxidizing condition the rate of phosphotransfer reduces to nominal. Under oxidizing condition the disulfide bond formed between C108–C261, that tethers large lid domain with the αβα domain, would hinder the release of F6P from the active site (Fig. 3b). Blocking the release of F6P would affect the coupled reaction and NADH formation thereby showing nominal rate. Reduction of the disulfide bridge, however, regains the activity. Therefore, our results suggest that closing and reopening of the lid during fructose binding or F6P release is essential for fructokinase activity.

### Structural basis of allosteric activation by monovalent cation

**Figure 4:**
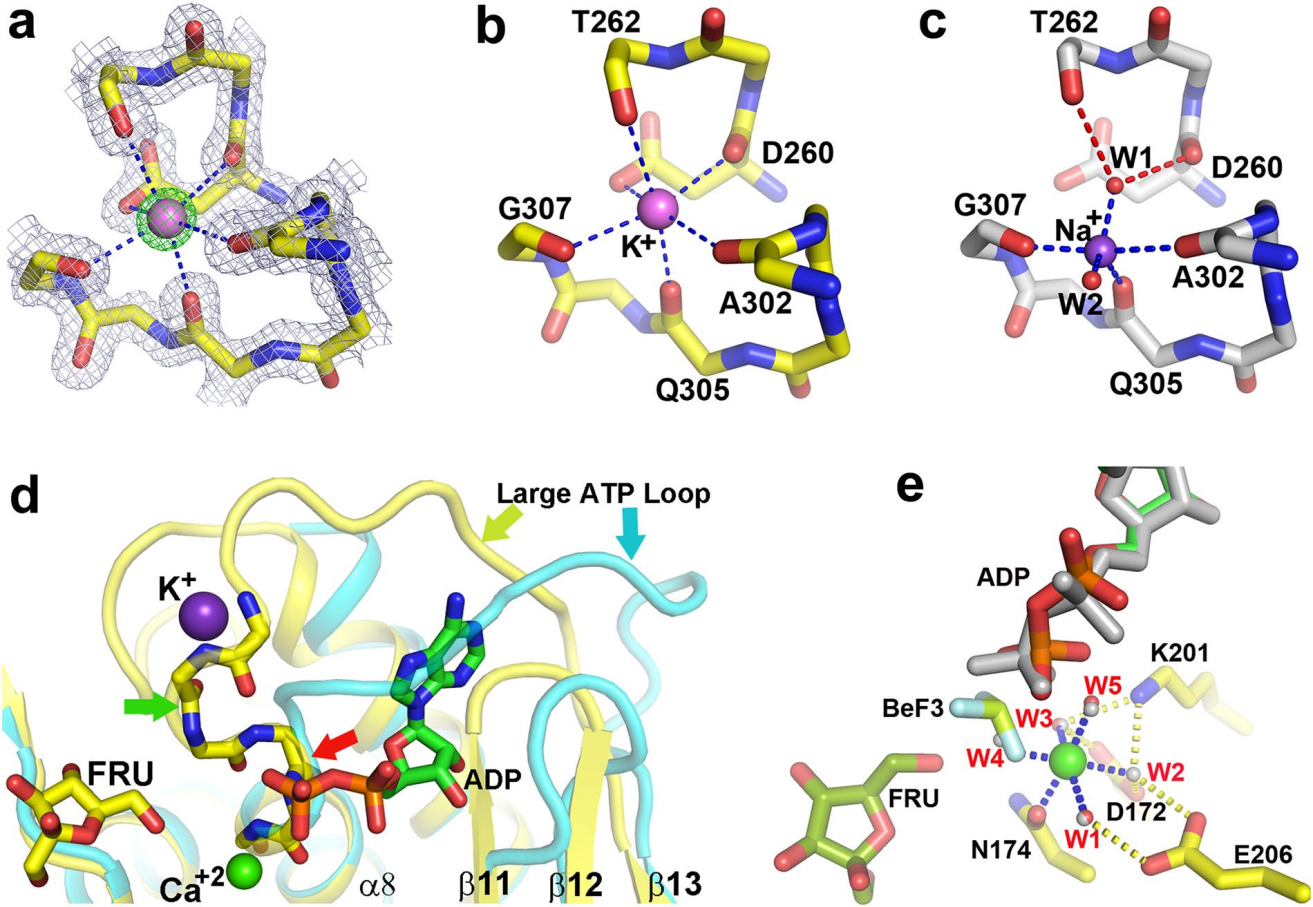
Mono/di valent cations in activation and catalysis of *Vc*FRK. (**a**) *2F*_*o*_ − *F*_*c*_ electron density map (slate mesh; 1.5σ) around K^+^ (pink ball) binding residues overlaid with *F*_*o*_*-F*_*c*_ map (green mesh; 15σ) confirming the position of K^+^. (**b**) Coordination of K^+^ (blue dash) with large ATP loop residues and metal binding loop. (**c**) Na^+^ ion (violet ball) coordination (blue dash) with metal binding loop and W1 and W2 (red balls). W1 connects large ATP loop with Na^+^ (red dash). (**d**) Comparison of activated (yellow) and non-activated *Vc*FRK (cyan); K^+^ (violet sphere), ADP, Ca^2+^ and fructose are shown for clarity. Main-chain of ^261^TTGAGD^266^ is shown as sticks, flipping of T262 carbonyl oxygen towards metal is indicated (green arrow). Point of deviation of large ATP loop in apo structure (red arrow) and its occlusion of ATP binding site is evident. (**e**) Divalent metal binding seen in fructose-ADP-Ca^2+^ (grey) and fructose-ADP-BeF_3_^−^-Ca^2+^ bound *Vc*FRK structure. Residues involved in metal binding (through water), ADP, fructose and BeF_3_^−^ are labeled.

RK family of sugar kinases is allosterically activated by K^+^/Cs^+^ ^19^ but the atomistic details of allosteric activation are largely unknown. In this study, we provide a complete structural basis of activation by comparing *Vc*FRK structures without metal ion, and *Vc*FRK bound to Na^+^/K^+^. This comparison is expected to provide a clearer picture of activation mechanism since apo and different monovalent metal bound kinases being compared here are from the same source. During activation by K^+^ (Fig. 4a, b), the metal ion occupies a position where it can simultaneously bind the carbonyl oxygens (D260, T262) of the large ATP loop and (A302, Q305, G307) of monovalent metal binding loop in an octahedral geometry (Fig. 4b). The average distance between two collinear axial oxygen atoms in *Vc*FRK is ~5.5 Å which matches closely with other RK family activated structures. This distance fits well for K^+^, NH_4_^+^^20^ but too big for Na^+^ and little small for Cs^+^. Apo *Vc*FRK crystallized with two molecules in the asymmetric unit and an inspection of the electron density map of chain A indicated a positive density at 4σ near the monovalent cation binding site. This density looks somewhat similar to a water molecule, but careful inspection of the disposition of the neighboring polar atoms and their distances from this peak indicated a pentavalent coordination which impelled us to assign this density as Na^+^, the only cation used during crystallization. Interestingly, crystal structure of Na^+^ ion activated *Vc*FRK looks very similar to K^+^ activated structure, raising a question why Na^+^ is a poor activator? Mode of coordination of Na^+^ ion provides the basis for its inefficient activation. Na^+^ ion is too small to simultaneously coordinate the carbonyl oxygens of the large ATP loop and the metal binding loop unlike K^+^ does. Rather, it interacts only with the carbonyl oxygens of the metal binding loop and assumes a penta-coordination state with two water molecules W1 and W2 (Fig. 4c). Interactions of Na^+^ with the large ATP loop are mediated through W2 and therefore, the interactions are weak (~2.8 Å). Kinetic experiments also exhibit maximum kinase activity in the presence of K^+^, followed by Cs^+^ (Fig. 3c) and no activity was observed for Na^+^ upto 200 mM NaCl, which substantiate our crystallographic observations.

Comparison of apo and activated *Vc*FRK structures shows that in apo form the large ATP loop is disorganized whereas the metal binding loop and α9 could not be located in electron density (Fig. 4d). Moreover, large ATP loop in apo form encroaches the ATP binding pocket, whereas in activated structure N-terminal part of this loop folds into an extra helical turn. As a result, remaining part of the loop is pulled away from the ATP binding site thereby removing the steric hindrance for incoming ATP (Fig. 4d). Formation of the helical turn not only pulls the loop and removes steric hindrance for incoming ATP it also assists to house the γ-phosphate of ATP in two ways. First, the carbonyl oxygen of the peptide bond between T262–G263 (preceding the conserved ^263^GAGD^266^ motif) is pulled towards K^+^, making a ‘nest’ within the helical structure that can house the γ-phosphate group. Secondly, due to this flipping amide nitrogens of four residues G263–D266 become exposed thereby providing it a favorable environment for the γ-phosphate (Fig. 4d). As judged from the location of BeF_3_^−^ in the *Vc*FRK-fructose-ADP-BeF_3_^−^ structure or modeling the γ-phosphate using *Vc*FRK-fructose-ADP structure as template, it is clear that in the unflipped form this carbonyl oxygen would cause severe contacts with the γ-phosphate. Thus flipping of this particular carbonyl oxygen is a key step in the activation of *Vc*FRK to provide appropriate space and environment to the γ-phosphate.

### ADP binding and role of divalent cations

No density for Ca^2+^ is observed in apo or sugar bound structure although it was present in the crystallization buffer. However, clear electron density for Ca^2+^ is consistently observed in both the *Vc*FRK-fructose-ADP and *Vc*FRK-fructose-ADP-BeF_3_^−^ structures indicating that ADP/ATP assist Ca^2+^ binding. The adenine moiety of ADP molecule binds in a shallow groove in between the small ATP loop and the large ATP loop (Fig. 1a) through weak hydrogen bonds with the main-chain O of V256 whereas its ribose part binds with the carbonyl oxygen of G239 and the OD2 atom of N296. There is almost no structural change upon ADP binding except the main chain of the loop connecting β11–β12, moves about 1.8 Å (Fig. 4d). The α-phosphate group of ADP does not make any direct H-bonds with *Vc*FRK, its β-phosphate interacts with the main chain N atoms of the ‘anion hole’.

In *Vc*FRK-fructose-ADP complex, Ca^2+^ binds with four equatorial water molecules (W1–W4) whereas OD1 atom of N174 and W5 serves as two almost collinear axial ligands. In *Vc*FRK-fructose-ADP-BeF_3_^−^ complex, W4 is replaced by a fluorine atom of BeF_3_^−^ (Fig. 4e). Ca^2+^ coordinating waters (W1-W5) are tightly held (B-factors ~10 Å^2^) by D172, K201 and E206, three highly conserved residues of FRK family (Figs 1e and 4e). There is no direct contact between divalent cation and ADP, in both the cases, but a weak interaction is seen through W5 (Fig. 4e). The only direct interaction of Ca^2+^ with *Vc*FRK is through the OD1 atom of N174, a highly conserved residue in FRK family. Interestingly, loop harboring N174 is about 10 residues longer due to an insertion between β9 and α5 in *Vc*FRK. This is not seen in *Ec*RK and is a unique feature in *Vc*FRK. Lid domains of *Vc*FRK are of comparable length, whereas lid domain (β2-β3) is 10 residues longer in *Ec*RK and its tip suffers a perpendicular bend to form the β-clasp^5^. This tip region is topologically equivalent to the loop connecting β9 and α5 that harbors two key residues N174 and R176 implicated to bind the divalent metal and fructose.

### *Vc*FRK-Fructose-ADP-BeF_3_^−^-Ca^2+^ structure resembles a Michaelis complex

To date, no significant report is available for the transition state intermediate of sugar phosphorylation^17,21^. Atomic-resolution structure of *Vc*FRK-fructose-ADP-BeF_3_^−^-Ca^2+^ is the first instance of a productively bound BeF_3_^−^ sugar kinase complex. One of the intriguing facts of this structure is that BeF_3_^−^ has been trapped in between β-phosphate of ADP and O6′ of fructose (Fig. 4e). Tetrahedral Be^2+^ atom remains 2.5 Å away from O2B of ADP (2.8 Å from the P atom of β-phosphate) and 3 Å away from O6′ of fructose, which are greater than P-O bond distance. Moreover, O2B of ADP, Be^2+^ and O6′ of fructose are seen almost in-line for phosphoryl transfer. One fluorine atom of BeF_3_^−^ is coordinated with the Ca^2+^ while the other two interacts with the ‘anion hole’ and R176 implying the role of divalent metal and anion hole in stabilization of γ-phosphate during phosphotransfer. In case of Mg^2+^, BeF_3_^−^ is expected to be pulled further towards metal ion and O2B of ADP, Be^2+^ and O6′ of fructose would be in-line for better phosphotransfer as seen in Fig. 3d.

### Implication of negatively charged surface around ATP on phosphorylation

**Figure 5:**
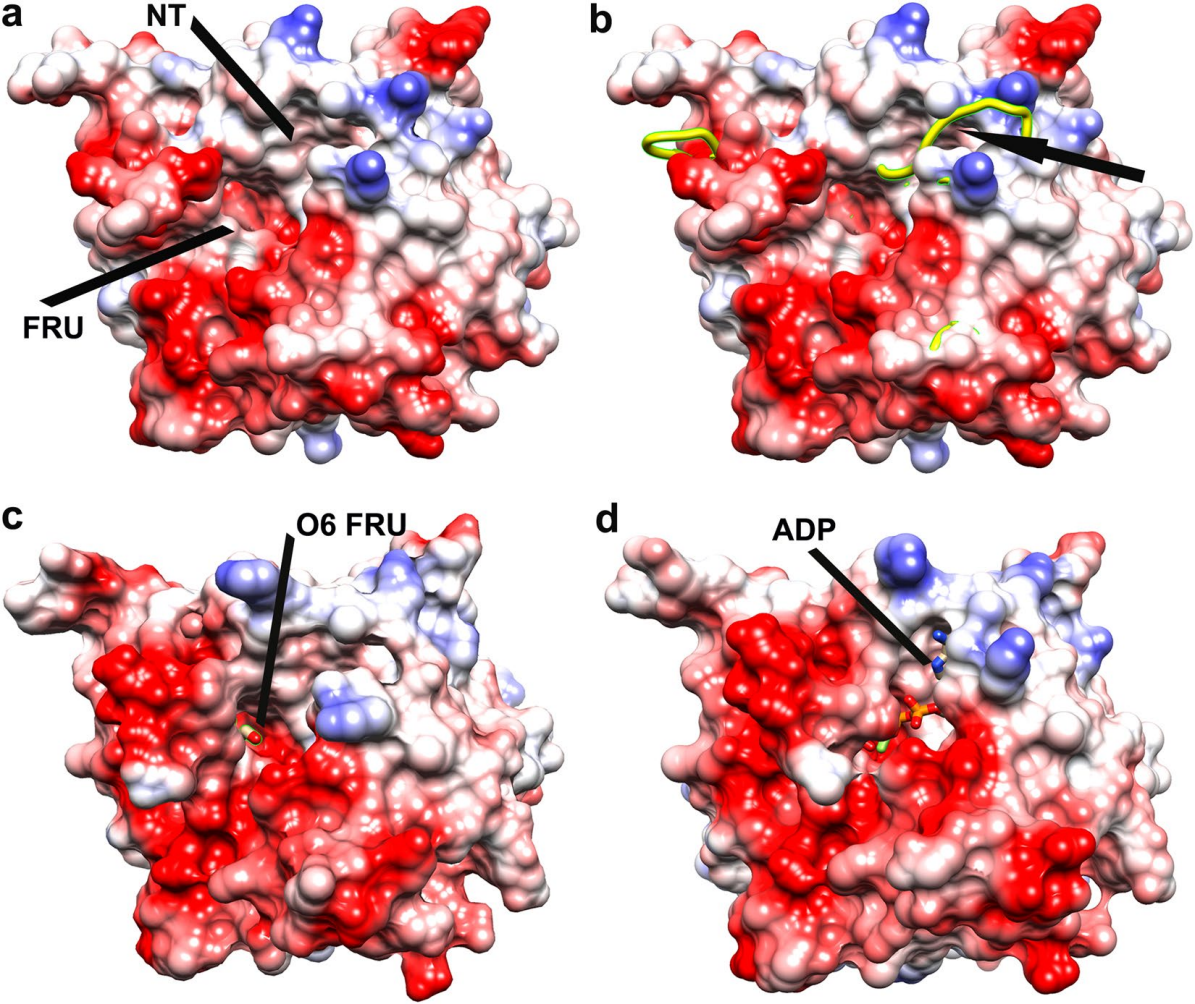
Change in surface grooves and electrostatic charge upon activation and sugar/ADP-BeF_3_^-^ binding of *Vc*FRK. (**a**) Monovalent cation bound *Vc*FRK where binding sites for fructose and ATP are indicated. (**b**) Superposition of monovalent cation bound (in surface) and apo (in yellow ribbon) *Vc*FRK showing large ATP loop of apo *Vc*FRK encroaches ATP binding site (black arrow). (**c**) Fructose bound *Vc*FRK where all but O6 atom of fructose is buried. Negative charge redistribution around sugar is evident when compared with (**a**). (**d**) Fructose-ADP-BeF_3_^-^ bound structure where β phosphate of ADP, BeF_3_^-^ and divalent metal are shielded from solvent.

Members of ribokinase superfamily utilize ATP or other nucleotide triphosphate to phosphorylate their cognate sugars. Like most other members of this family, sugar binding site in *Vc*FRK is a pre-formed groove located at the junction of the lid domain and the αβα domain (Fig. 5a). The ATP binding site primarily forms upon monovalent cation binding (Figs 4d and 5b) which is fine tuned after fructose binding (Fig. 5c). Chemical nature of ATP renders the terminal triphosphate group flexible amid different possible conformations. Not all conformations of ATP are directed towards the pertinent alcohol group of fructose for productive phosphorylation. Therefore, built-in mechanism to redirect the γ-phosphate would certainly increase the rate of phosphotransfer. Fructose binding in *Vc*FRK redistributes highly conserved E110, E204, E205 and E206 around the phosphate groups of ATP (Fig. 5c). In Apo structure, these residues remain far away but approach towards ADP/ADP-BeF_3_^−^ upon fructose binding. Conformations of ATP facing these negative charges are expected to be repelled and adopt low barrier alternate conformations facing fructose. This electrostatic entrapment of ATP is essential for the fidelity of the phosphotransfer reaction. We prepared two *Vc*FRK mutants E110Q and E205Q, having identical bulk and shape but differing in charge, to see the efficiency of this electrostatic entrapment. Both the mutants showed more than 100 times decreased activity, compared to the wild type, supporting this view.

## Discussions

In addition to glycolysis, fructose metabolism is a critical component of cellular metabolism. FRK, a member of PfkB family, catalyzes the conversion of fructose to F6P, which is metabolized into dihydroxyacetone phosphate and glyceraldehyde by aldolase^18^ and subsequently converges in the glycolytic pathway^14^. In general, the common structural features of PfkB family members comprise of a single αβα domain and they oligomerize to create an active site at the interface of two subunits^7,22^. Besides αβα domain few members of the PfkB family contain a flexible lid sub-domain formed by two antiparallel β-strands. *Vc*FRK along with *Ec*RK, AIR, KDG kinase^23,24^ and 1O14 (deposited in the Protein Data Bank; PDB CODE 2AJR) belongs to this group and they all homodimerize by lid-to-lid interaction, although their mode of dimerization is quite different. Lid domains interact to form a β-clasp type of structure in *Ec*RK or 1O14 while in *Vc*FRK, AIR and KDG kinases they formm a side-by-side or flap kind of structure. All members of PfkB family posses a conserved aspartic acid believed to act as a base to encourage deprotonation of the pertinent sugar hydroxyl group to be phosphorylated. However, residues around this aspartic acid and their disposition vary significantly in different subfamilies to efficiently bind their cognate sugar. In general, these residues form extensive hydrogen bonds to sequester the sugar and immobilize it before phosphorylation. Moreover, their typical arrangement helps to discriminate among different anomeric forms of a sugar or preferentially bind the same anomeric form but in different orientation depending upon which hydroxyl group of sugar would be phosphorylated. In *Vc*FRK fructose sequestering residues are optimally oriented to H-bond with all the polar atoms of fructose preferentially in β-D-furanose form.

**Figure 6:**
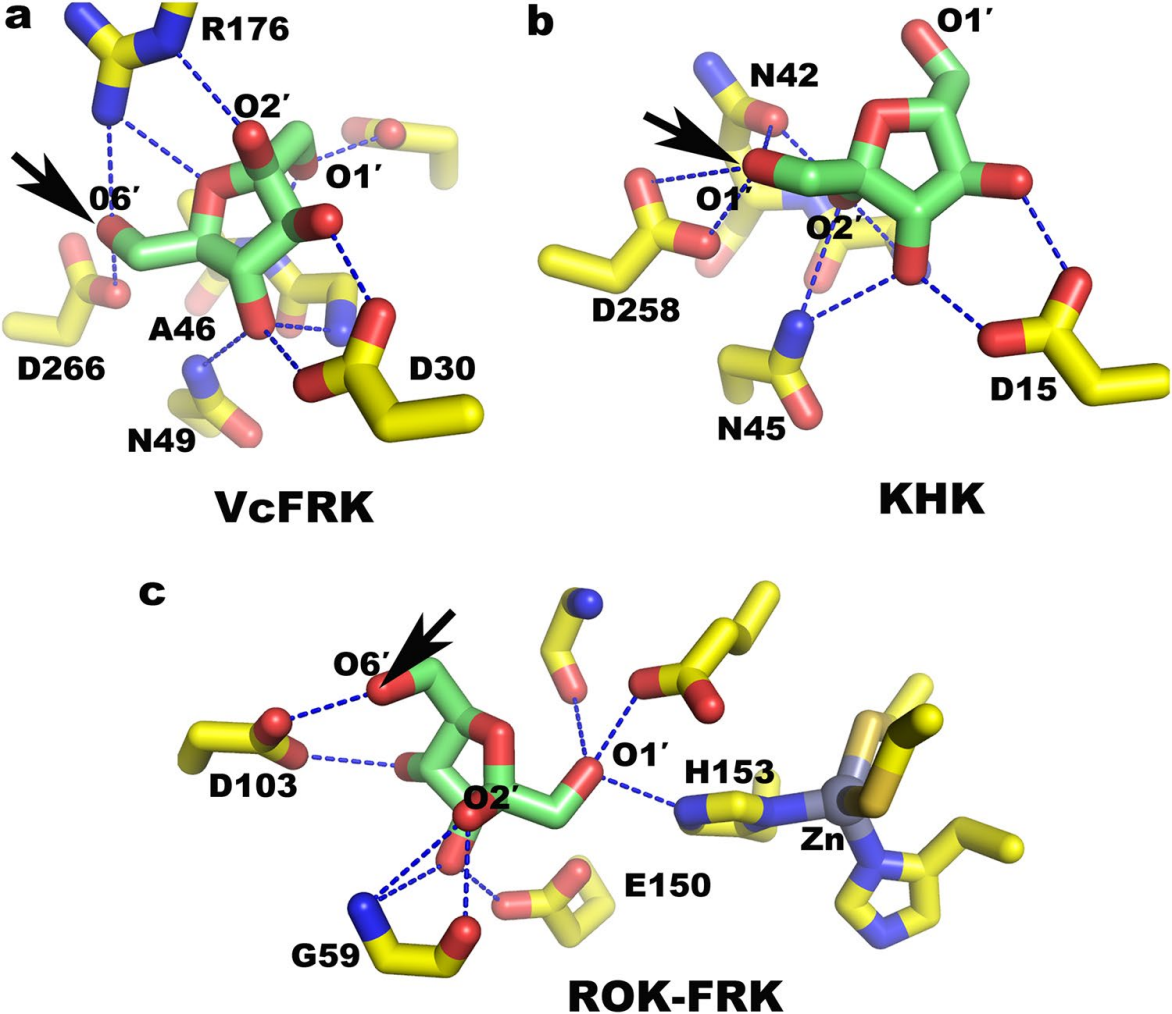
Active site design to bind fructose in a preferred orientation. (**a**) O6′ atom of fructose is poised to attack ATP in *Vc*FRK, crucial residues stabilizing this orientation are labeled. (**b**) O1′ atom of fructose is orientated to attack ATP in KHK (PDB code 2HW1) residues crucial to bind fructose in this preferred orientation are labeled and (**c**) phosphorylation of fructose at O6′ atom in ROK-FRK (PDB code 3LM9). Residues crucial to bind in this orientation and 2His-2Cys-Zn cluster close to the sugar binding site are shown. D266, D258 and D103 that deprotonate the pertinent sugar hydroxyl group in *Vc*FRK, KHK and ROK-FRK respectively occupy identical position. Stabilization of O2′ in binding sugar in a preferred orientation is evident; site of phosphorylation is shown in black arrow.

Fructose is phosphorylated either at its O1′ position by Ketohexokinase (KHK) or O6′ atom by FRK to yield F1P and F6P, respectively. To maintain this specificity, it is customary to selectively bind the fructose and orient either its O1′ atom or O6′ atom, as the case may be, poised to attack the γ-phosphate. *Vc*FRK and KHK have some crucial architectural difference at their fructose binding site that support preferential binding of fructose in one orientation over the other. Interaction of fructose with the active site residues, particularly its O2′, is a crucial determinant in binding fructose in a particular orientation. In *Vc*FRK, O2′ of fructose binds with R176 which is absent in KHK while in KHK O2′ of fructose H-bonded with N42 unlike Ala (A46) in *Vc*FRK (Fig. 6a, b). Therefore, phosphorylation of fructose at O1′ by FRK or at O6′ by KHK would not be feasible. Again, fructose binding site of ROK-FRK and *Vc*FRK is totally different. Here G59 plays a crucial role in stabilizing the O2′ atom so much so that G59A mutation displays nominal fructokinase activity (Fig. 6c). D103, E150 of ROK-FRK roughly corresponds to D266, D30 of *Vc*FRK (Fig. 6a) or D258, D15 of KHK (Fig. 6b). But a 2His-2Cys-Zn cluster is unique in ROK-FRK which is located close to the sugar binding site and the metal ion has been implicated in allosteric control of the reaction (Fig. 6c).

In *Vc*FRK, huge movement (>20 Å) of the large lid sub-domain occurs upon sequestration of fructose (Fig. 2a). Side-by-side mode of dimerization in *Vc*FRK, with less involvement of large lid sub-domain in dimerization, makes it possible. Consequently, binding of fructose has little impact on the overall dimeric shape and dimension (Fig. 1b). β-clasp mode of dimerization in *Ec*RK involve both the lid domains with intricate H-bonds with its dimeric partner. Therefore, sugar binding and associated movements are coupled resulting in huge changes in overall dimension^17^. Movement of the large lid domain is required in *Vc*FRK to bind and sequester the sugar as long as it is not phosphorylated but needs to reopen during F6P release. Consistent closure/opening of the large lid sub-domain is therefore essential for the activity of *Vc*FRK. Arresting the movement by tethering the large lid domain to the αβα domain impairs catalytic activity of *Vc*FRK.

Allosteric activation by K^+^/Cs^+^ induces another significant structural change leading to *Vc*FRK enzyme activity. Structural comparison between S. *aureus* apo RK, and activated *Ec*RK^25^ showed a disorganized large ATP loop which occludes the ATP binding pocket. However, this study could not shed light on atomic details of activation, formation of anion hole and preference for K^+^/Cs^+^. Since the apo and activated structures used in this comparison are from different sources, delineation of detail atom-by-atom comparison would be overestimated. Our structural study not only demonstrate the detailed mechanism of allosteric activation but also provide the structural basis why K^+^/Cs^+^ is preferred in activation over Na^+^/Li^+^. The average distance (~5.5 Å) between two collinear octahedrally coordinating axial ligands, in RK family of enzymes, is a crucial determinant. This distance fits well for K^+^, NH_4_^+^ making K^+^ as preferred physiological activator.

**Figure 7:**
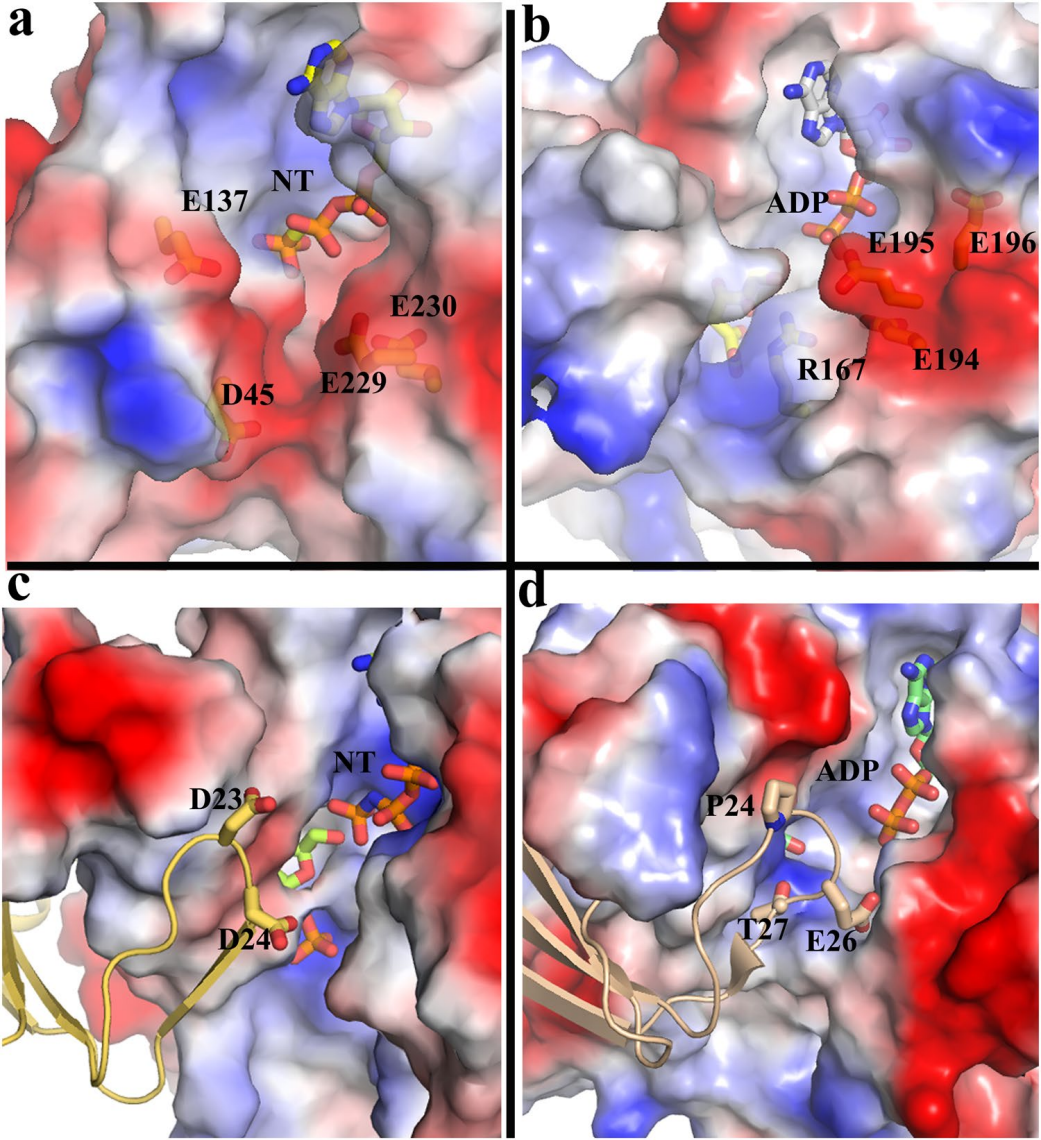
Redirection of phosphate group by negative charge in other sugar kinases. (**a**) Adenosine kinase (PDB code 1LII) and (**b**) tagatose 6-phosphate kinase (PDB code 2JG1) where residues from the same monomer engaged to form the negative patch. In dimeric (**c**) KDGK (PDB code 1V1A) and (**d**) ribokinase (PDB code 4XDA) β-clasp mode of dimerization where negative charge(s) at the lid loop reinforce with the negative charge around ATP of the other protomer.

Productive neucleophilic attack, requires positioning of ATP γ-phosphate in close proximity of fructose O6′ atom. Chemical nature of ATP renders the terminal triphosphate group highly flexible amid different possible conformations. Not all conformations direct the γ-phosphate of ATP towards O6 atom of fructose for productive phosphorylation. Redistribution of several glutamic acid residues upon sugar binding has been envisaged to play a key role in redirecting those conformations of ATP towards fructose. These glutamic acids are stubborn conserve in other FRKs implying similar electrostatic entrapment mechanism of ATP is operative for those FRKs. Mutants E110Q and E205Q, having identical bulk and shape but differing in charge, showed less than 100 times activity compared to the wild type supporting this view. Presence of similar negatively charged environment in several other RK sub-family members strengthens our view. The simplest of them is Adenosine kinase^26^ working as a monomer where a negatively charged patch consisting of highly conserved residues is identified around ATP (Fig. 7a). Similar negatively charged environment is also found in tagatose 6-phosphate kinase where residues E194, E195 and E196 are contributed to form the negative patch^27^ (Fig. 7b). RK^28^ and 2-Keto-3-deoxygluconate kinase^24^ form β-clasp mode of dimerization which disposes the tip of lid domain of one monomer close to the active site of the other (Fig. 7c,d). Negatively charged residue(s) at the lid domain merges with the negative charges around ATP of the other protomer and together they redirect the γ-phosphate group towards sugar.

In the activated sugar and ATP bound *Vc*FRK, deprotonated O6′ atom of fructose undergoes nucleophilic attack on the γ-phosphate of ATP and generate the transition state intermediate. Atomic-resolution structure of *Vc*FRK-fructose-ADP-BeF_3_^−^-Ca^2+^ provide the exact location of the tetrahedral BeF_3_^−^. Location of the divalent metal is such that it further limits the conformational space of β/γ-phosphates in between Ca^2+^ and the ‘anion hole’. This situation is ideal to facilitate in-line transfer of phosphate. In the transition state, BeF_3_^−^ is expected to be planner and could still be stabilized by the anion hole, divalent metal. R176 can adopt conformations to stabilize the planner γ-phosphate in the transition state. Moreover, upon BeF_3_^−^ binding, H108 flips and interacts with the α-phosphate while conserved G109 allows large lid domain to come in close to ATP and screens it from solvent thereby reducing unwanted hydrolysis of ATP. However, upon phosphoryl transfer, phosphate group of F6P would be too close to N174 and D266 and the resulting repulsive force would collapse the tethering interactions and facilitate F6P release. The alternate egress of sugar is already blocked due to monovalent cation binding. Altogether our data indicate that catalytic transfer of γ-phosphate to fructose is governed by the ligand assisted cumulative changes of *Vc*FRK along the reaction coordinates. Kinetic data of VcFRK and its mutants, provided here, substantiate the mechanism proposed based on structural observations.

## Methods

Unless otherwise stated, all chemicals and general reagents were purchased from Sigma-Aldrich.

### Cloning, expression and purification

The *CscK* gene encoding *Vc*FRK was cloned, over-expressed and purified as reported earlier^29^. *Vc*FRK mutants E110Q and E205Q were cloned by two-step PCR method. Both wild type and mutant proteins were over-expressed as N-terminal 6 × His tag and purified by Ni-NTA affinity chromatography followed by Thrombin cleavage and gel filtration chromatography.

### Size exclusion chromatography (SEC) of *Vc*FRK

Oligomeric states of *Vc*FRK were analyzed by size exclusion chromatography on a ÄKTAPrime chromatographic systems using Superdex 200 increase (GE-Healthcare) column (10 × 300 mm) pre-equilibrated with lysis buffer (50 mM Tris-HCl pH 8.0, 300 mM NaCl, 20 mM KCl containing 0.02% sodium azide) running at 10 °C with a flow rate of 1.0 ml min^−1^. The elution profile was determined by monitoring the absorbance at 280 nm. Bovine serum albumin, ovalbumin, chymotrypsinogen, and RNase A were used as molecular weight standards.

### Crystallization, data collection and structure solution

Crystallization and data collection of apo *Vc*FRK (2.46 Å) and *Vc*FRK+fructose+ADP (1.75 Å) were reported earlier^29^. *Vc*FRK+fructose and *Vc*FRK+fructose+ADP+BeF_3_^−^, *Vc*FRK-DM+ADP+Fructose+Ca^2+^+K^+^ crystals were grown later and they diffract to a resolution of 2.4 Å, 1.3 Å and 1.7 Å, respectively. Data for *Vc*FRK+fructose+ADP+BeF_3_^−^ was collected at beamline BM14, European Synchrotron Radiation Facility (ESRF), Grenoble, France. All other datasets were collected at home source. At first, the structure of *Vc*FRK+fructose+ADP (1.75 Å) was solved because of its superior data quality. Subsequently other structures were solved using the coordinates of *Vc*FRK+fructose+ADP. The coordinates of *Se*AIRSK^9^ was used for molecular replacement with Phaser^30^ in CCP4^31^. Two molecule of the search model produced a TFZ = 16.7 and LLG = 531. Model building was done with Coot^32^ and refinement was carried out with Phenix refine^33^. TLS refinement was performed during the final stages^33^. In each case, 5% of the reflections were used to calculate the R_free_ value. Statistics on data collection and structural refinements are given in Table 1. Sodium, potassium and calcium ions were placed on the basis of their electron density map and coordination distances.

### Structural analysis

B-factors and interchain/intrachain interactions were calculated using B_average_ and CONTACT program in CCP4^31^. The oligomeric state was analyzed using PISA webserver^15^. Sequence analysis was done using Multalin^34^. Figures were prepared using Pymol (http://www.pymol.org) and chimera^35^.

### Isothermal titration calorimetry (ITC)

ITC experiments were performed using ITC 200 instrument (Microcal, GE Healthcare) at 25 °C by injecting ligand loaded into the injection syringe titrated against purified protein loaded into the sample cell. Runs consisted of a 500:1 ligand:protein ratio. All solutions were pre-dialyzed in buffer to mitigate effects of dilution and degassed to avoid air bubbles in the calorimeter during the experiment. Data used to calculate binding constants were referenced against runs performed with ligand alone to control for the heat of ligand solvation. The sample cell was filled with 0.04 mM protein solution and the injection syringe was filled with 20 mM sugar in 20 mM Tris (pH 8.0), 100 mM NaCl, 20 mM KCl. The reaction was monitored for binding study with injections of 2 µl each for 20 injections having 120 sec of interval between them at 25 °C. All the data were corrected for the heat of dilution produced by continued injection of ligand into the reaction cell. The data was fitted using ORIGIN software.

### Enzyme activity assays

Fructokinase activity was measured according to the coupled assay^18^ using UV-VIS spectrophotometry (JASCO) at 340 nm by detecting the reduced Nicotinamide Adenine Dinucleotide (NAD^+^, ε_340_ = 6.22 cm^−1^ mM^−1^) at 25 °C in an assay buffer [50 mM Tris-HCl (MERCK) pH 8.0, 0.5 mM KCl (SRL), 0.5 mM NAD^+^, 0.5 mM ATP, 0.5 mM MgCl_2_ (SRL), 0.025 mM-1.0 mM fructose, 1.0 units/ml glucose-6-phosphate dehydrogenase, and 1.0 unit/ml phosphoglucose isomerase]. The reaction was initiated by the addition of 0.014 μM enzyme, and the absorbance at 340 nm was measured after 10 minutes. To check the tethering effect of *Vc*FRK-DM, the protein was first incubated with fructose and then the disulphide bond formation was induced by adding equimolar amount of H_2_O_2_. The mixture was reduced with β-mercapto ethanol to remove the tethering. Enzymatic activity was expressed as millimole of substrate converted per minute per micromole of enzyme. The K_m_ and V_max_ values against ATP were obtained by fitting to Lineweaver-Burk equation. Three independent experiments were carried out and mean values were used for analysis.

## Electronic supplementary material

Supplementary Information
