# Supplementary material for: Large-scale conformational changes and redistribution of surface negative charge upon sugar binding dictate the fidelity of phosphorylation in Vibrio cholerae fructokinase
Source: Sci Rep. 2018 Nov 16;8:16925. doi: 10.1038/s41598-018-35236-3 (PMC6240065; doi:10.1038/s41598-018-35236-3)
Supplement: Supplementary file 1 — Supplementary Information [file 41598_2018_35236_MOESM1_ESM.pdf]

Supplementary Information

Large-scale conformational changes and redistribution of surface negative charge upon sugar binding dictate the fidelity of phosphorylation in *Vibrio cholerae* fructokinase

Rakhi Paul, Shramana Chatterjee, Seema Nath and Udayaditya Sen*

Crystallography and Molecular Biology Division,

Saha Institute of Nuclear Physics, HBNI, 1/AF Bidhan Nagar, Kolkata 700064, India.

*To whom correspondence should be addressed: Crystallography and Molecular Biology Division, Saha Institute of Nuclear Physics, 1/AF Bidhan Nagar, Kolkata 700 064, India. Tel: 91-33-2337-5345-49;

Fax: 91-33-2337-4637 E-Mail: udayaditya.sen@saha.ac.in;

Index

Sl. No.	Content	Page
1	Supplemental Figure 1: Dimeric VcFRK in solution.	3
2	PDB X-ray Structure Validation Report of 5EY7	4
3	PDB X-ray Structure Validation Report of 5EYN	17
4	PDB X-ray Structure Validation Report of 5F0Z	31
5	PDB X-ray Structure Validation Report of 5YGG	44
6	PDB X-ray Structure Validation Report of 5F11	58

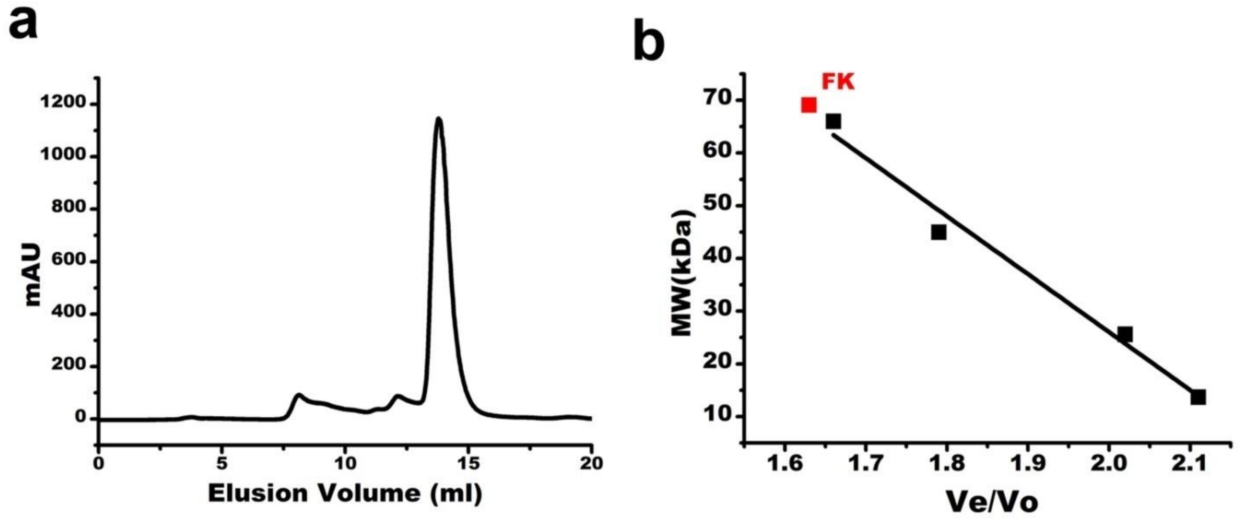

Supplemental Figure 1. Dimeric VcFRK in solution. (a) Elution profile of VcFRK in Superdex-200 size-exclusion column. (b) Elution volume of VcFRK (large peak) corresponds to a dimeric species as calculated from a standard curve.

Full wwPDB X-ray Structure Validation Report (i)

Aug 7, 2017 – 09:53 PM EDT

PDB ID	:	5EY7
Title	:	Crystal structure of Fructokinase from Vibrio cholerae O395 in apo form
Authors	:	Paul, R.; Nath, S.; Sen, U.
Deposited on	:	unknown
Resolution	:	2.46 Å(reported)

This is a Full wwPDB X-ray Structure Validation Report for a publicly released PDB entry.

We welcome your comments at *validation@mail.wwpdb.org*
A user guide is available at
http://wwpdb.org/validation/2016/XrayValidationReportHelp
with specific help available everywhere you see the (i) symbol.

The following versions of software and data (see references (i)) were used in the production of this report:

MolProbity	:	4.02b-467
Xtriage (Phenix)	:	1.9-1692
EDS	:	rb-20029824
Percentile statistics	:	20161228.v01 (using entries in the PDB archive December 28th 2016)
Refmac	:	5.8.0135
CCP4	:	6.5.0
Ideal geometry (proteins)	:	Engh & Huber (2001)
Ideal geometry (DNA, RNA)	:	Parkinson et al. (1996)
Validation Pipeline (wwPDB-VP)	:	rb-20029824

1 Overall quality at a glance (i)

The following experimental techniques were used to determine the structure:
X-RAY DIFFRACTION

The reported resolution of this entry is 2.46 Å.

Percentile scores (ranging between 0-100) for global validation metrics of the entry are shown in the following graphic. The table shows the number of entries on which the scores are based.

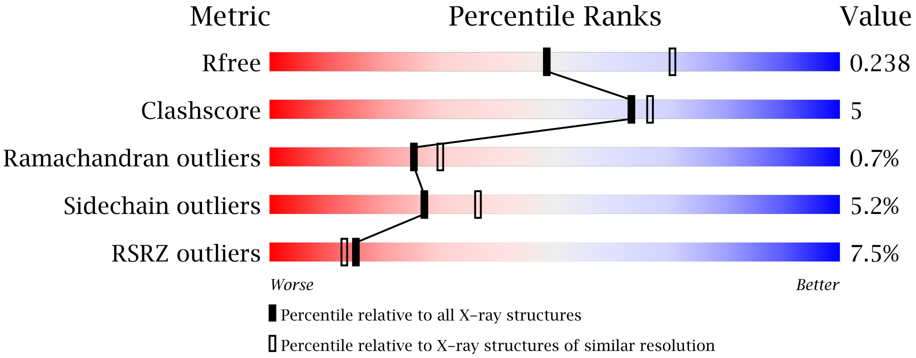

Metric	Whole archive (#Entries)	Similar resolution (#Entries, resolution range(Å))
R_{free}	100719	1119 (2.48-2.44)
Clashscore	112137	1193 (2.48-2.44)
Ramachandran outliers	110173	1185 (2.48-2.44)
Sidechain outliers	110143	1185 (2.48-2.44)
RSRZ outliers	101464	1126 (2.48-2.44)

The table below summarises the geometric issues observed across the polymeric chains and their fit to the electron density. The red, orange, yellow and green segments on the lower bar indicate the fraction of residues that contain outliers for >=3, 2, 1 and 0 types of geometric quality criteria. A grey segment represents the fraction of residues that are not modelled. The numeric value for each fraction is indicated below the corresponding segment, with a dot representing fractions <=5% The upper red bar (where present) indicates the fraction of residues that have poor fit to the electron density. The numeric value is given above the bar.

Mol	Chain	Length	Quality of chain
1	A	323	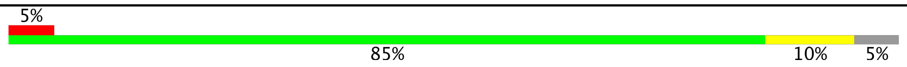
1	B	323	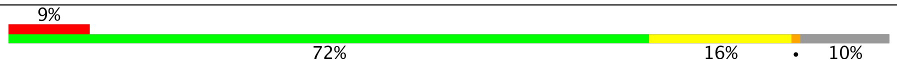

2 Entry composition (i)

There are 3 unique types of molecules in this entry. The entry contains 4627 atoms, of which 0 are hydrogens and 0 are deuteriums.

In the tables below, the ZeroOcc column contains the number of atoms modelled with zero occupancy, the AltConf column contains the number of residues with at least one atom in alternate conformation and the Trace column contains the number of residues modelled with at most 2 atoms.

- Molecule 1 is a protein called Fructokinase.

Mol	Chain	Residues	Atoms					ZeroOcc	AltConf	Trace
			Total	C	N	O	S			
1	A	307	2332	1475	400	447	10	0	0	0
1	B	291	2211	1396	382	424	9	0	0	0

- Molecule 2 is SODIUM ION (three-letter code: NA) (formula: Na).

Mol	Chain	Residues	Atoms		ZeroOcc	AltConf
			Total	Na		
2	A	1	1	1	0	0

- Molecule 3 is water.

Mol	Chain	Residues	Atoms		ZeroOcc	AltConf
			Total	O		
3	A	47	47	47	0	0
3	B	36	36	36	0	0

3 Residue-property plots

These plots are drawn for all protein, RNA and DNA chains in the entry. The first graphic for a chain summarises the proportions of the various outlier classes displayed in the second graphic. The second graphic shows the sequence view annotated by issues in geometry and electron density. Residues are color-coded according to the number of geometric quality criteria for which they contain at least one outlier: green = 0, yellow = 1, orange = 2 and red = 3 or more. A red dot above a residue indicates a poor fit to the electron density (RSRZ > 2). Stretches of 2 or more consecutive residues without any outlier are shown as a green connector. Residues present in the sample, but not in the model, are shown in grey.

- Molecule 1: Fructokinase

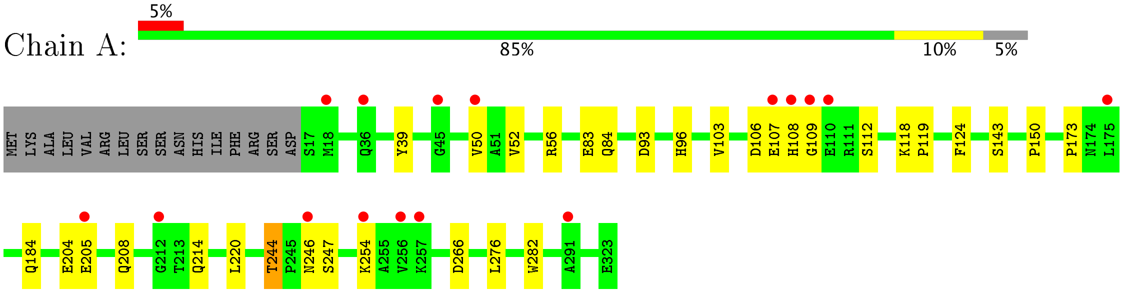

- Molecule 1: Fructokinase

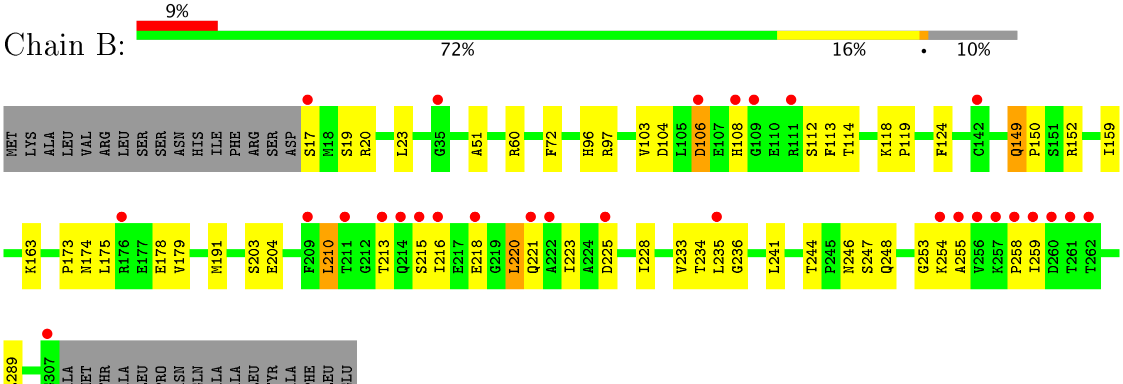

4 Data and refinement statistics (i)

Property	Value	Source
Space group	P 21 21 2	Depositor
Cell constants<br>a, b, c, α, β, γ	107.12Å 99.86Å 61.55Å<br>90.00° 90.00° 90.00°	Depositor
Resolution (Å)	29.41 – 2.46<br>29.41 – 2.46	Depositor<br>EDS
% Data completeness<br>(in resolution range)	95.0 (29.41-2.46)<br>91.2 (29.41-2.46)	Depositor<br>EDS
R_{merge}	0.06	Depositor
R_{sym}	(Not available)	Depositor
$< I/\sigma(I) >$ [1]	2.75 (at 2.48Å)	Xtriage
Refinement program	PHENIX 1.9_1692	Depositor
R, R_{free}	0.215 , 0.242<br>0.214 , 0.238	Depositor<br>DCC
R_{free} test set	1126 reflections (5.02%)	DCC
Wilson B-factor (Å^2)	41.7	Xtriage
Anisotropy	0.872	Xtriage
Bulk solvent k_{sol}(e/Å^3), B_{sol}(Å^2)	0.31 , 48.7	EDS
L-test for twinning[2]	$< \|L\| >$ = 0.50, $< L^2 >$ = 0.33	Xtriage
Estimated twinning fraction	No twinning to report.	Xtriage
F_o,F_c correlation	0.94	EDS
Total number of atoms	4627	wwPDB-VP
Average B, all atoms (Å^2)	69.0	wwPDB-VP

Xtriage's analysis on translational NCS is as follows: *The largest off-origin peak in the Patterson function is 8.97% of the height of the origin peak. No significant pseudotranslation is detected.*

[1]Intensities estimated from amplitudes.

[2]Theoretical values of $< |L| >$, $< L^2 >$ for acentric reflections are 0.5, 0.333 respectively for untwinned datasets, and 0.375, 0.2 for perfectly twinned datasets.

5 Model quality (i)

5.1 Standard geometry (i)

Bond lengths and bond angles in the following residue types are not validated in this section: NA

The Z score for a bond length (or angle) is the number of standard deviations the observed value is removed from the expected value. A bond length (or angle) with $|Z| > 5$ is considered an outlier worth inspection. RMSZ is the root-mean-square of all Z scores of the bond lengths (or angles).

Mol	Chain	Bond lengths		Bond angles	
		RMSZ	#|Z| >5	RMSZ	#|Z| >5
1	A	0.26	0/2382	0.43	0/3240
1	B	0.25	0/2258	0.41	0/3071
All	All	0.25	0/4640	0.42	0/6311

There are no bond length outliers.

There are no bond angle outliers.

There are no chirality outliers.

There are no planarity outliers.

5.2 Too-close contacts (i)

In the following table, the Non-H and H(model) columns list the number of non-hydrogen atoms and hydrogen atoms in the chain respectively. The H(added) column lists the number of hydrogen atoms added and optimized by MolProbity. The Clashes column lists the number of clashes within the asymmetric unit, whereas Symm-Clashes lists symmetry related clashes.

Mol	Chain	Non-H	H(model)	H(added)	Clashes	Symm-Clashes
1	A	2332	0	2283	18	0
1	B	2211	0	2164	27	0
2	A	1	0	0	0	0
3	A	47	0	0	0	0
3	B	36	0	0	1	0
All	All	4627	0	4447	44	0

The all-atom clashscore is defined as the number of clashes found per 1000 atoms (including hydrogen atoms). The all-atom clashscore for this structure is 5.

All (44) close contacts within the same asymmetric unit are listed below, sorted by their clash magnitude.

Atom-1	Atom-2	Interatomic distance (Å)	Clash overlap (Å)
1:A:106:ASP:HB2	1:A:112:SER:HB3	1.60	0.83
1:B:213:THR:HG21	1:B:218:GLU:HB2	1.77	0.65
1:A:56:ARG:NH1	1:A:83:GLU:OE2	2.30	0.64
1:B:218:GLU:HA	1:B:221:GLN:HB3	1.78	0.64
1:A:244:THR:HG22	1:A:247:SER:H	1.63	0.63
1:A:93:ASP:OD2	1:A:96:HIS:ND1	2.29	0.62
1:B:244:THR:HG22	1:B:246:ASN:H	1.64	0.61
1:B:223:ILE:HD13	1:B:228:ILE:HD12	1.81	0.61
1:A:276:LEU:HD22	1:A:282:TRP:HB2	1.83	0.60
1:A:83:GLU:N	1:A:84:GLN:HA	2.17	0.60
1:A:143:SER:HB3	1:A:173:PRO:HA	1.84	0.59
1:B:103:VAL:HG12	1:B:113:PHE:HB3	1.84	0.59
1:B:60:ARG:NH1	3:B:402:HOH:O	2.32	0.58
1:B:104:ASP:N	1:B:112:SER:O	2.30	0.54
1:A:83:GLU:H	1:A:84:GLN:HA	1.74	0.53
1:B:149:GLN:HG3	1:B:150:PRO:HA	1.91	0.53
1:A:124:PHE:HA	1:A:150:PRO:HD2	1.91	0.52
1:B:96:HIS:O	1:B:97:ARG:NH1	2.43	0.52
1:A:52:VAL:HG22	1:A:83:GLU:HG2	1.94	0.50
1:A:108:HIS:N	1:A:109:GLY:HA2	2.26	0.50
1:B:235:LEU:HD11	1:B:241:LEU:HB2	1.94	0.49
1:A:107:GLU:O	1:A:108:HIS:HB2	2.13	0.48
1:A:208:GLN:NE2	1:A:214:GLN:O	2.46	0.48
1:B:118:LYS:HA	1:B:119:PRO:HA	1.80	0.47
1:B:124:PHE:HA	1:B:150:PRO:HD2	1.96	0.46
1:B:233:VAL:HB	1:B:241:LEU:HB3	1.97	0.46
1:B:106:ASP:HB3	1:B:108:HIS:H	1.81	0.46
1:B:173:PRO:O	1:B:175:LEU:N	2.49	0.45
1:B:17:SER:HB2	1:B:60:ARG:CZ	2.48	0.44
1:B:253:GLY:O	1:B:255:ALA:N	2.51	0.43
1:A:244:THR:HG23	1:A:246:ASN:H	1.84	0.43
1:A:39:TYR:HE2	1:B:72:PHE:HE1	1.65	0.43
1:B:213:THR:C	1:B:215:SER:H	2.22	0.43
1:B:103:VAL:HA	1:B:113:PHE:HA	2.00	0.43
1:A:50:VAL:HG22	1:A:266:ASP:O	2.20	0.42
1:B:213:THR:CG2	1:B:218:GLU:HB2	2.48	0.41
1:B:216:ILE:HG13	1:B:216:ILE:H	1.67	0.41
1:A:118:LYS:HA	1:A:119:PRO:HA	1.74	0.41
1:B:159:ILE:O	1:B:163:LYS:HG3	2.20	0.41
1:A:52:VAL:HG13	1:A:83:GLU:HG2	2.03	0.41
1:B:220:LEU:O	1:B:223:ILE:HG13	2.20	0.41
1:B:23:LEU:HD13	1:B:51:ALA:HA	2.02	0.41

Continued on next page...

Continued from previous page...

Atom-1	Atom-2	Interatomic distance (Å)	Clash overlap (Å)
1:B:216:ILE:O	1:B:220:LEU:HB2	2.20	0.40
1:B:191:MET:SD	1:B:210:LEU:HG	2.62	0.40

There are no symmetry-related clashes.

5.3 Torsion angles (i)

5.3.1 Protein backbone (i)

In the following table, the Percentiles column shows the percent Ramachandran outliers of the chain as a percentile score with respect to all X-ray entries followed by that with respect to entries of similar resolution.

The Analysed column shows the number of residues for which the backbone conformation was analysed, and the total number of residues.

Mol	Chain	Analysed	Favoured	Allowed	Outliers	Percentiles	
1	A	305/323 (94%)	292 (96%)	13 (4%)	0	100	100
1	B	289/323 (90%)	262 (91%)	23 (8%)	4 (1%)	13	12
All	All	594/646 (92%)	554 (93%)	36 (6%)	4 (1%)	25	30

All (4) Ramachandran outliers are listed below:

Mol	Chain	Res	Type
1	B	254	LYS
1	B	258	PRO
1	B	174	ASN
1	B	236	GLY

5.3.2 Protein sidechains (i)

In the following table, the Percentiles column shows the percent sidechain outliers of the chain as a percentile score with respect to all X-ray entries followed by that with respect to entries of similar resolution.

The Analysed column shows the number of residues for which the sidechain conformation was analysed, and the total number of residues.

Continued on next page...

Continued from previous page...

Mol	Chain	Analysed	Rotameric	Outliers	Percentiles	
1	A	247/262 (94%)	240 (97%)	7 (3%)	49	63
1	B	236/262 (90%)	218 (92%)	18 (8%)	15	19
All	All	483/524 (92%)	458 (95%)	25 (5%)	27	36

All (25) residues with a non-rotameric sidechain are listed below:

Mol	Chain	Res	Type
1	A	103	VAL
1	A	184	GLN
1	A	204	GLU
1	A	205	GLU
1	A	220	LEU
1	A	244	THR
1	A	254	LYS
1	B	19	SER
1	B	20	ARG
1	B	106	ASP
1	B	114	THR
1	B	149	GLN
1	B	152	ARG
1	B	178	GLU
1	B	179	VAL
1	B	203	SER
1	B	204	GLU
1	B	210	LEU
1	B	220	LEU
1	B	225	ASP
1	B	234	THR
1	B	247	SER
1	B	248	GLN
1	B	259	ILE
1	B	289	LEU

Some sidechains can be flipped to improve hydrogen bonding and reduce clashes. All (2) such sidechains are listed below:

Mol	Chain	Res	Type
1	A	249	GLN
1	A	285	GLN

5.3.3 RNA (i)

There are no RNA molecules in this entry.

5.4 Non-standard residues in protein, DNA, RNA chains (i)

There are no non-standard protein/DNA/RNA residues in this entry.

5.5 Carbohydrates (i)

There are no carbohydrates in this entry.

5.6 Ligand geometry (i)

Of 1 ligands modelled in this entry, 1 is monoatomic - leaving 0 for Mogul analysis.

There are no bond length outliers.

There are no bond angle outliers.

There are no chirality outliers.

There are no torsion outliers.

There are no ring outliers.

No monomer is involved in short contacts.

5.7 Other polymers (i)

There are no such residues in this entry.

5.8 Polymer linkage issues (i)

There are no chain breaks in this entry.

6 Fit of model and data (i)

6.1 Protein, DNA and RNA chains (i)

In the following table, the column labelled '#RSRZ> 2' contains the number (and percentage) of RSRZ outliers, followed by percent RSRZ outliers for the chain as percentile scores relative to all X-ray entries and entries of similar resolution. The OWAB column contains the minimum, median, 95th percentile and maximum values of the occupancy-weighted average B-factor per residue. The column labelled 'Q< 0.9' lists the number of (and percentage) of residues with an average occupancy less than 0.9.

Mol	Chain	Analysed	<RSRZ>	#RSRZ>2			OWAB(Å^2)	Q<0.9
1	A	307/323 (95%)	0.27	16 (5%)	28	26	36, 60, 112, 178	0
1	B	291/323 (90%)	0.56	29 (9%)	8	6	37, 63, 135, 158	0
All	All	598/646 (92%)	0.41	45 (7%)	15	13	36, 62, 127, 178	0

All (45) RSRZ outliers are listed below:

Mol	Chain	Res	Type	RSRZ
1	B	259	ILE	9.2
1	B	258	PRO	7.2
1	B	261	THR	5.9
1	B	35	GLY	5.7
1	B	262	THR	4.8
1	B	209	PHE	4.4
1	B	17	SER	4.1
1	A	108	HIS	4.1
1	B	214	GLN	3.9
1	B	254	LYS	3.8
1	B	255	ALA	3.8
1	A	18	MET	3.7
1	B	221	GLN	3.7
1	B	213	THR	3.7
1	B	218	GLU	3.7
1	A	109	GLY	3.7
1	B	211	THR	3.7
1	A	256	VAL	3.4
1	B	142	CYS	3.4
1	B	257	LYS	3.3
1	A	107	GLU	3.2
1	A	36	GLN	3.2
1	B	260	ASP	3.1
1	A	257	LYS	3.0

Continued on next page...

Continued from previous page...

Mol	Chain	Res	Type	RSRZ
1	B	307	GLY	2.9
1	B	225	ASP	2.8
1	B	216	ILE	2.8
1	B	222	ALA	2.7
1	A	110	GLU	2.7
1	B	108	HIS	2.7
1	B	176	ARG	2.7
1	B	256	VAL	2.6
1	B	235	LEU	2.6
1	A	254	LYS	2.5
1	B	215	SER	2.5
1	B	109	GLY	2.5
1	A	246	ASN	2.5
1	A	50	VAL	2.4
1	A	175	LEU	2.4
1	B	106	ASP	2.1
1	A	291	ALA	2.1
1	A	205	GLU	2.1
1	A	45	GLY	2.1
1	A	212	GLY	2.1
1	B	111	ARG	2.0

6.2 Non-standard residues in protein, DNA, RNA chains (i)

There are no non-standard protein/DNA/RNA residues in this entry.

6.3 Carbohydrates (i)

There are no carbohydrates in this entry.

6.4 Ligands (i)

In the following table, the Atoms column lists the number of modelled atoms in the group and the number defined in the chemical component dictionary. LLDF column lists the quality of electron density of the group with respect to its neighbouring residues in protein, DNA or RNA chains. The B-factors column lists the minimum, median, 95th percentile and maximum values of B factors of atoms in the group. The column labelled 'Q< 0.9' lists the number of atoms with occupancy less than 0.9.

Mol	Type	Chain	Res	Atoms	RSCC	RSR	LLDF	B-factors(Å^2)	Q<0.9
2	NA	A	401	1/1	0.96	0.16	-0.20	79,79,79,79	0

6.5 Other polymers (i)

There are no such residues in this entry.

Full wwPDB X-ray Structure Validation Report (i)

Feb 13, 2017 – 01:09 am GMT

PDB ID	:	5EYN
Title	:	Crystal structure of Fructokinase from Vibrio cholerae O395 in fructose, ADP, Beryllium trifluoride and calcium ion bound form
Authors	:	Paul, R.; Nath, S.; Sen, U.
Deposited on	:	2015-11-25
Resolution	:	1.29 Å(reported)

This is a Full wwPDB X-ray Structure Validation Report for a publicly released PDB entry.

We welcome your comments at *validation@mail.wwpdb.org*
A user guide is available at
http://wwpdb.org/validation/2016/XrayValidationReportHelp
with specific help available everywhere you see the (i) symbol.

The following versions of software and data (see references (i)) were used in the production of this report:

MolProbity	:	4.02b-467
Mogul	:	1.7.2 (RC1), CSD as538be (2017)
Xtriage (Phenix)	:	1.9-1692
EDS	:	trunk28620
Percentile statistics	:	20161228.v01 (using entries in the PDB archive December 28th 2016)
Refmac	:	5.8.0135
CCP4	:	6.5.0
Ideal geometry (proteins)	:	Engh & Huber (2001)
Ideal geometry (DNA, RNA)	:	Parkinson et al. (1996)
Validation Pipeline (wwPDB-VP)	:	recalc28949

1 Overall quality at a glance (i)

The following experimental techniques were used to determine the structure:
X-RAY DIFFRACTION

The reported resolution of this entry is 1.29 Å.

Percentile scores (ranging between 0-100) for global validation metrics of the entry are shown in the following graphic. The table shows the number of entries on which the scores are based.

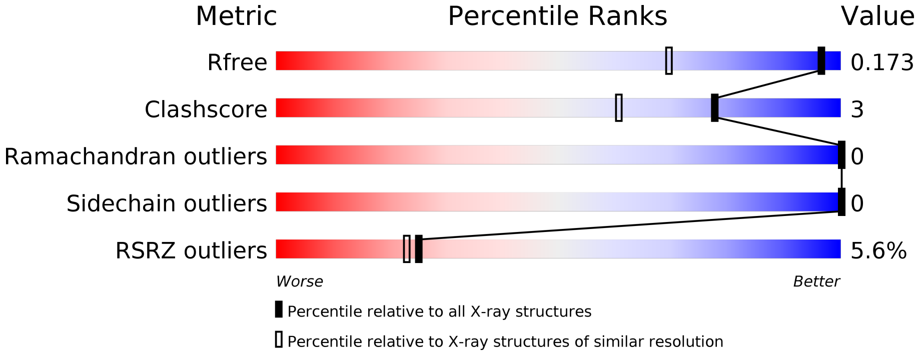

Metric	Whole archive (#Entries)	Similar resolution (#Entries, resolution range(Å))
R_{free}	100719	1367 (1.30-1.26)
Clashscore	112137	1447 (1.30-1.26)
Ramachandran outliers	110173	1392 (1.30-1.26)
Sidechain outliers	110143	1391 (1.30-1.26)
RSRZ outliers	101464	1370 (1.30-1.26)

The table below summarises the geometric issues observed across the polymeric chains and their fit to the electron density. The red, orange, yellow and green segments on the lower bar indicate the fraction of residues that contain outliers for >=3, 2, 1 and 0 types of geometric quality criteria. A grey segment represents the fraction of residues that are not modelled. The numeric value for each fraction is indicated below the corresponding segment, with a dot representing fractions <=5% The upper red bar (where present) indicates the fraction of residues that have poor fit to the electron density. The numeric value is given above the bar.

Mol	Chain	Length	Quality of chain
1	A	323	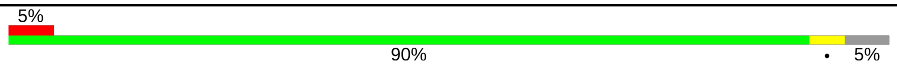

The following table lists non-polymeric compounds, carbohydrate monomers and non-standard residues in protein, DNA, RNA chains that are outliers for geometric or electron-density-fit criteria:

Mol	Type	Chain	Res	Chirality	Geometry	Clashes	Electron density
4	BEF	A	403	-	-	X	X
6	NA	A	405	-	-	-	X

2 Entry composition (i)

There are 7 unique types of molecules in this entry. The entry contains 5059 atoms, of which 2310 are hydrogens and 0 are deuteriums.

In the tables below, the ZeroOcc column contains the number of atoms modelled with zero occupancy, the AltConf column contains the number of residues with at least one atom in alternate conformation and the Trace column contains the number of residues modelled with at most 2 atoms.

- Molecule 1 is a protein called Fructokinase.

Mol	Chain	Residues	Atoms						ZeroOcc	AltConf	Trace
			Total	C	H	N	O	S			
1	A	306	4611	1472	2286	399	444	10	0	0	0

- Molecule 2 is FRUCTOSE (three-letter code: FRU) (formula: $C_6H_{12}O_6$).

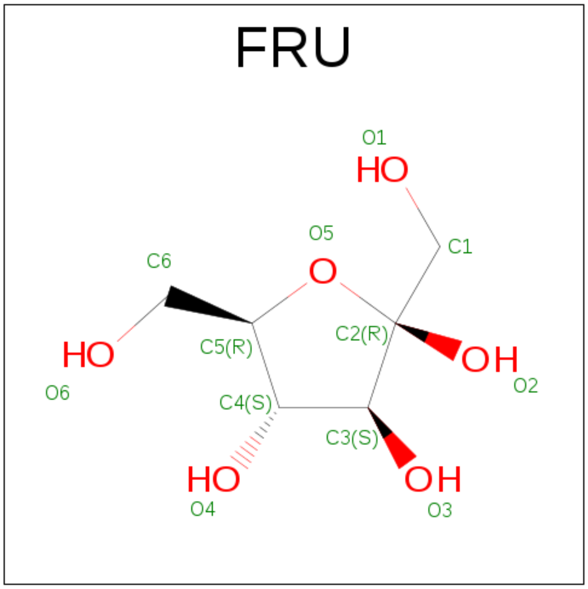

Mol	Chain	Residues	Atoms				ZeroOcc	AltConf
			Total	C	H	O		
2	A	1	24	6	12	6	0	0

- Molecule 3 is CALCIUM ION (three-letter code: CA) (formula: Ca).

Mol	Chain	Residues	Atoms		ZeroOcc	AltConf
			Total	Ca		
3	A	1	1	1	0	0

- Molecule 4 is BERYLLIUM TRIFLUORIDE ION (three-letter code: BEF) (formula: BeF_3).

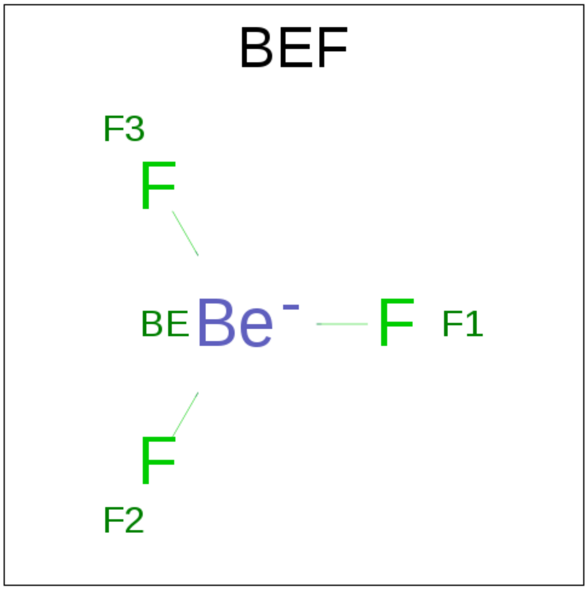

Mol	Chain	Residues	Atoms			ZeroOcc	AltConf
			Total	Be	F		
4	A	1	4	1	3	0	0

- Molecule 5 is ADENOSINE-5'-DIPHOSPHATE (three-letter code: ADP) (formula: $C_{10}H_{15}N_5O_{10}P_2$).

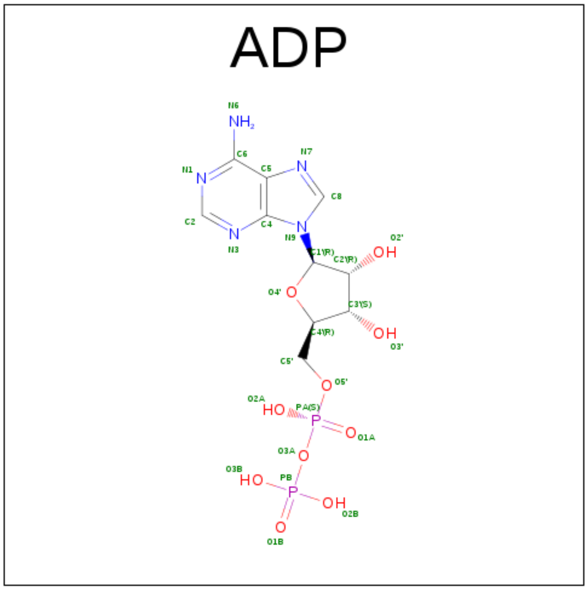

Mol	Chain	Residues	Atoms						ZeroOcc	AltConf
			Total	C	H	N	O	P		
5	A	1	39	10	12	5	10	2	0	0

- Molecule 6 is SODIUM ION (three-letter code: NA) (formula: Na).

Mol	Chain	Residues	Atoms		ZeroOcc	AltConf
			Total	Na		
6	A	1	1	1	0	0

- Molecule 7 is water.

Mol	Chain	Residues	Atoms		ZeroOcc	AltConf
			Total	O		
7	A	379	379	379	0	0

3 Residue-property plots (i)

These plots are drawn for all protein, RNA and DNA chains in the entry. The first graphic for a chain summarises the proportions of the various outlier classes displayed in the second graphic. The second graphic shows the sequence view annotated by issues in geometry and electron density. Residues are color-coded according to the number of geometric quality criteria for which they contain at least one outlier: green = 0, yellow = 1, orange = 2 and red = 3 or more. A red dot above a residue indicates a poor fit to the electron density (RSRZ > 2). Stretches of 2 or more consecutive residues without any outlier are shown as a green connector. Residues present in the sample, but not in the model, are shown in grey.

- Molecule 1: Fructokinase

Chain A:

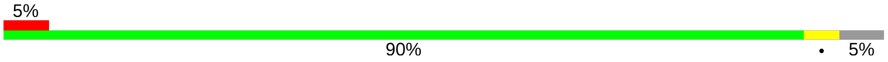

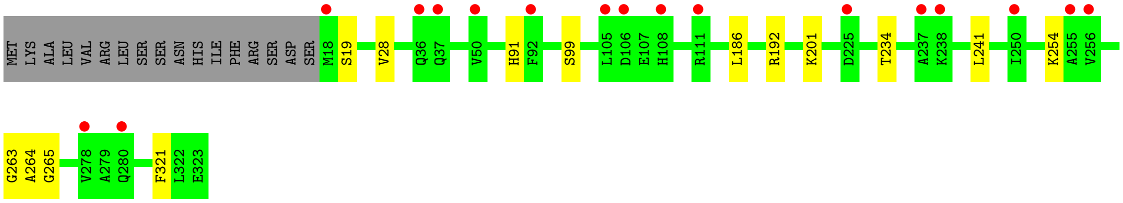

4 Data and refinement statistics (i)

Property	Value	Source
Space group	P 21 21 2	Depositor
Cell constants<br>a, b, c, α, β, γ	64.30Å 107.22Å 41.62Å<br>90.00° 90.00° 90.00°	Depositor
Resolution (Å)	32.15 – 1.29<br>32.15 – 1.29	Depositor<br>EDS
% Data completeness<br>(in resolution range)	98.0 (32.15-1.29)<br>98.1 (32.15-1.29)	Depositor<br>EDS
R_{merge}	0.04	Depositor
R_{sym}	(Not available)	Depositor
$< I/\sigma(I) >$ [1]	2.39 (at 1.29Å)	Xtriage
Refinement program	PHENIX (phenix.refine: 1.8_1069)	Depositor
R, R_{free}	0.141 , 0.169<br>0.148 , 0.173	Depositor<br>DCC
R_{free} test set	3615 reflections (5.29%)	DCC
Wilson B-factor (Å^2)	15.7	Xtriage
Anisotropy	0.607	Xtriage
Bulk solvent k_{sol}(e/Å^3), B_{sol}(Å^2)	0.47 , 50.3	EDS
L-test for twinning[2]	$< \|L\| > = 0.49$, $< L^2 > = 0.32$	Xtriage
Estimated twinning fraction	No twinning to report.	Xtriage
F_o,F_c correlation	0.98	EDS
Total number of atoms	5059	wwPDB-VP
Average B, all atoms (Å^2)	16.0	wwPDB-VP

Xtriage's analysis on translational NCS is as follows: *The largest off-origin peak in the Patterson function is 5.79% of the height of the origin peak. No significant pseudotranslation is detected.*

[1] Intensities estimated from amplitudes.

[2] Theoretical values of $< |L| >$, $< L^2 >$ for acentric reflections are 0.5, 0.333 respectively for untwinned datasets, and 0.375, 0.2 for perfectly twinned datasets.

5 Model quality (i)

5.1 Standard geometry (i)

Bond lengths and bond angles in the following residue types are not validated in this section: NA, CA, BEF, FRU, ADP

The Z score for a bond length (or angle) is the number of standard deviations the observed value is removed from the expected value. A bond length (or angle) with $|Z| > 5$ is considered an outlier worth inspection. RMSZ is the root-mean-square of all Z scores of the bond lengths (or angles).

Mol	Chain	Bond lengths		Bond angles	
		RMSZ	#|Z| >5	RMSZ	#|Z| >5
1	A	0.39	0/2375	0.57	0/3232

There are no bond length outliers.

There are no bond angle outliers.

There are no chirality outliers.

There are no planarity outliers.

5.2 Too-close contacts (i)

In the following table, the Non-H and H(model) columns list the number of non-hydrogen atoms and hydrogen atoms in the chain respectively. The H(added) column lists the number of hydrogen atoms added and optimized by MolProbity. The Clashes column lists the number of clashes within the asymmetric unit, whereas Symm-Clashes lists symmetry related clashes.

Mol	Chain	Non-H	H(model)	H(added)	Clashes	Symm-Clashes
1	A	2325	2286	2278	12	0
2	A	12	12	12	0	0
3	A	1	0	0	0	0
4	A	4	0	0	4	0
5	A	27	12	12	6	0
6	A	1	0	0	0	0
7	A	379	0	0	5	3
All	All	2749	2310	2302	16	3

The all-atom clashscore is defined as the number of clashes found per 1000 atoms (including hydrogen atoms). The all-atom clashscore for this structure is 3.

All (16) close contacts within the same asymmetric unit are listed below, sorted by their clash magnitude.

Atom-1	Atom-2	Interatomic distance (Å)	Clash overlap (Å)
5:A:404:ADP:O2B	7:A:501:HOH:O	1.84	0.96
4:A:403:BEF:F1	5:A:404:ADP:O1B	2.02	0.68
1:A:19:SER:OG	7:A:502:HOH:O	2.13	0.65
1:A:91:HIS:CD2	7:A:507:HOH:O	2.53	0.61
4:A:403:BEF:F1	5:A:404:ADP:PB	2.49	0.60
1:A:265:GLY:H	5:A:404:ADP:PB	2.28	0.55
1:A:264:ALA:N	5:A:404:ADP:O1B	2.43	0.51
1:A:254:LYS:HG3	1:A:321:PHE:CZ	2.48	0.49
1:A:91:HIS:NE2	7:A:507:HOH:O	2.36	0.47
1:A:241:LEU:HD23	1:A:241:LEU:C	2.37	0.45
4:A:403:BEF:F2	5:A:404:ADP:O1B	2.26	0.45
1:A:201:LYS:HE2	1:A:234:THR:CG2	2.48	0.44
1:A:28:VAL:HG12	1:A:99:SER:HB3	2.01	0.42
1:A:192:ARG:NH1	7:A:511:HOH:O	2.43	0.41
1:A:186:LEU:HD23	1:A:186:LEU:C	2.42	0.40
1:A:263:GLY:HA2	4:A:403:BEF:F2	2.11	0.40

All (3) symmetry-related close contacts are listed below. The label for Atom-2 includes the symmetry operator and encoded unit-cell translations to be applied.

Atom-1	Atom-2	Interatomic distance (Å)	Clash overlap (Å)
7:A:778:HOH:O	7:A:788:HOH:O[1_556]	2.12	0.08
7:A:514:HOH:O	7:A:864:HOH:O[1_556]	2.12	0.08
7:A:840:HOH:O	7:A:848:HOH:O[1_554]	2.19	0.01

5.3 Torsion angles (i)

5.3.1 Protein backbone (i)

In the following table, the Percentiles column shows the percent Ramachandran outliers of the chain as a percentile score with respect to all X-ray entries followed by that with respect to entries of similar resolution.

The Analysed column shows the number of residues for which the backbone conformation was analysed, and the total number of residues.

Mol	Chain	Analysed	Favoured	Allowed	Outliers	Percentiles
1	A	304/323 (94%)	300 (99%)	4 (1%)	0	100 100

There are no Ramachandran outliers to report.

5.3.2 Protein sidechains (i)

In the following table, the Percentiles column shows the percent sidechain outliers of the chain as a percentile score with respect to all X-ray entries followed by that with respect to entries of similar resolution.

The Analysed column shows the number of residues for which the sidechain conformation was analysed, and the total number of residues.

Mol	Chain	Analysed	Rotameric	Outliers	Percentiles	
1	A	246/262 (94%)	246 (100%)	0	100	100

There are no protein residues with a non-rotameric sidechain to report.

Some sidechains can be flipped to improve hydrogen bonding and reduce clashes. All (1) such sidechains are listed below:

Mol	Chain	Res	Type
1	A	134	GLN

5.3.3 RNA (i)

There are no RNA molecules in this entry.

5.4 Non-standard residues in protein, DNA, RNA chains (i)

There are no non-standard protein/DNA/RNA residues in this entry.

5.5 Carbohydrates (i)

There are no carbohydrates in this entry.

5.6 Ligand geometry (i)

Of 5 ligands modelled in this entry, 2 are monoatomic - leaving 3 for Mogul analysis.

In the following table, the Counts columns list the number of bonds (or angles) for which Mogul statistics could be retrieved, the number of bonds (or angles) that are observed in the model and the number of bonds (or angles) that are defined in the chemical component dictionary. The Link column lists molecule types, if any, to which the group is linked. The Z score for a bond length (or angle) is the number of standard deviations the observed value is removed from the expected value. A bond length (or angle) with $|Z| > 2$ is considered an outlier worth inspection. RMSZ is the root-mean-square of all Z scores of the bond lengths (or angles).

Mol	Type	Chain	Res	Link	Bond lengths			Bond angles		
					Counts	RMSZ	#|Z| > 2	Counts	RMSZ	#|Z| > 2
2	FRU	A	401	-	11,12,12	1.11	1 (9%)	10,18,18	0.55	0
4	BEF	A	403	5	0,3,3	0.00	-	0,3,3	0.00	-
5	ADP	A	404	4	25,29,29	1.38	5 (20%)	24,45,45	2.35	4 (16%)

In the following table, the Chirals column lists the number of chiral outliers, the number of chiral centers analysed, the number of these observed in the model and the number defined in the chemical component dictionary. Similar counts are reported in the Torsion and Rings columns. '-' means no outliers of that kind were identified.

Mol	Type	Chain	Res	Link	Chirals	Torsions	Rings
2	FRU	A	401	-	-	0/5/24/24	0/1/1/1
4	BEF	A	403	5	-	0/0/0/0	0/0/0/0
5	ADP	A	404	4	-	0/12/32/32	0/3/3/3

All (6) bond length outliers are listed below:

Mol	Chain	Res	Type	Atoms	Z	Observed(Å)	Ideal(Å)
5	A	404	ADP	C2'-C1'	-3.51	1.48	1.53
2	A	401	FRU	O5-C2	-2.24	1.39	1.43
5	A	404	ADP	O2'-C2'	-2.08	1.38	1.43
5	A	404	ADP	C2'-C3'	-2.07	1.47	1.53
5	A	404	ADP	C2-N3	2.17	1.35	1.32
5	A	404	ADP	C6-N6	2.25	1.43	1.34

All (4) bond angle outliers are listed below:

Mol	Chain	Res	Type	Atoms	Z	Observed(^{o})	Ideal(^{o})
5	A	404	ADP	N3-C2-N1	-10.11	120.05	128.86
5	A	404	ADP	C4'-O4'-C1'	-2.98	106.60	109.77
5	A	404	ADP	C4-C5-N7	-2.32	107.17	109.41
5	A	404	ADP	C2-N1-C6	2.23	122.67	118.77

There are no chirality outliers.

There are no torsion outliers.

There are no ring outliers.

2 monomers are involved in 7 short contacts:

Mol	Chain	Res	Type	Clashes	Symm-Clashes
4	A	403	BEF	4	0
5	A	404	ADP	6	0

5.7 Other polymers (i)

There are no such residues in this entry.

5.8 Polymer linkage issues (i)

There are no chain breaks in this entry.

6 Fit of model and data (i)

6.1 Protein, DNA and RNA chains (i)

In the following table, the column labelled '#RSRZ> 2' contains the number (and percentage) of RSRZ outliers, followed by percent RSRZ outliers for the chain as percentile scores relative to all X-ray entries and entries of similar resolution. The OWAB column contains the minimum, median, 95th percentile and maximum values of the occupancy-weighted average B-factor per residue. The column labelled 'Q< 0.9' lists the number of (and percentage) of residues with an average occupancy less than 0.9.

Mol	Chain	Analysed	<RSRZ>	#RSRZ>2			OWAB(Å^2)	Q<0.9
1	A	306/323 (94%)	0.07	17 (5%)	25	23	6, 12, 25, 37	1 (0%)

All (17) RSRZ outliers are listed below:

Mol	Chain	Res	Type	RSRZ
1	A	18	MET	6.9
1	A	280	GLN	5.1
1	A	106	ASP	4.3
1	A	92	PHE	3.7
1	A	255	ALA	3.4
1	A	36	GLN	3.2
1	A	111	ARG	3.1
1	A	108	HIS	2.9
1	A	225	ASP	2.9
1	A	105	LEU	2.6
1	A	238	LYS	2.5
1	A	50	VAL	2.4
1	A	256	VAL	2.4
1	A	278	VAL	2.4
1	A	237	ALA	2.4
1	A	37	GLN	2.3
1	A	250	ILE	2.0

6.2 Non-standard residues in protein, DNA, RNA chains (i)

There are no non-standard protein/DNA/RNA residues in this entry.

6.3 Carbohydrates (i)

There are no carbohydrates in this entry.

6.4 Ligands (i)

In the following table, the Atoms column lists the number of modelled atoms in the group and the number defined in the chemical component dictionary. LLDF column lists the quality of electron density of the group with respect to its neighbouring residues in protein, DNA or RNA chains. The B-factors column lists the minimum, median, 95th percentile and maximum values of B factors of atoms in the group. The column labelled 'Q< 0.9' lists the number of atoms with occupancy less than 0.9.

Mol	Type	Chain	Res	Atoms	RSCC	RSR	LLDF	B-factors($Å^2$)	Q<0.9
4	BEF	A	403	4/4	0.74	0.22	6.26	14,17,38,62	0
6	NA	A	405	1/1	0.93	0.11	3.42	16,16,16,16	0
5	ADP	A	404	27/27	0.89	0.19	1.62	11,18,79,110	0
2	FRU	A	401	12/12	0.98	0.09	-0.25	6,7,9,10	0
3	CA	A	402	1/1	0.99	0.04	-2.65	18,18,18,18	0

6.5 Other polymers (i)

There are no such residues in this entry.

Full wwPDB X-ray Structure Validation Report ⓘ

Feb 13, 2017 – 01:05 am GMT

PDB ID	:	5F0Z
Title	:	Crystal structure of Fructokinase from Vibrio cholerae O395 in fructose, ADP and calcium ion bound form
Authors	:	Paul, R.; Nath, S.; Sen, U.
Deposited on	:	2015-11-28
Resolution	:	1.75 Å(reported)

This is a Full wwPDB X-ray Structure Validation Report for a publicly released PDB entry.

We welcome your comments at *validation@mail.wwpdb.org*
A user guide is available at
http://wwpdb.org/validation/2016/XrayValidationReportHelp
with specific help available everywhere you see the ⓘ symbol.

The following versions of software and data (see references ⓘ) were used in the production of this report:

MolProbity	:	4.02b-467
Mogul	:	1.7.2 (RC1), CSD as538be (2017)
Xtriage (Phenix)	:	1.9-1692
EDS	:	trunk28620
Percentile statistics	:	20161228.v01 (using entries in the PDB archive December 28th 2016)
Refmac	:	5.8.0135
CCP4	:	6.5.0
Ideal geometry (proteins)	:	Engh & Huber (2001)
Ideal geometry (DNA, RNA)	:	Parkinson et al. (1996)
Validation Pipeline (wwPDB-VP)	:	recalc28949

1 Overall quality at a glance (i)

The following experimental techniques were used to determine the structure:
X-RAY DIFFRACTION

The reported resolution of this entry is 1.75 Å.

Percentile scores (ranging between 0-100) for global validation metrics of the entry are shown in the following graphic. The table shows the number of entries on which the scores are based.

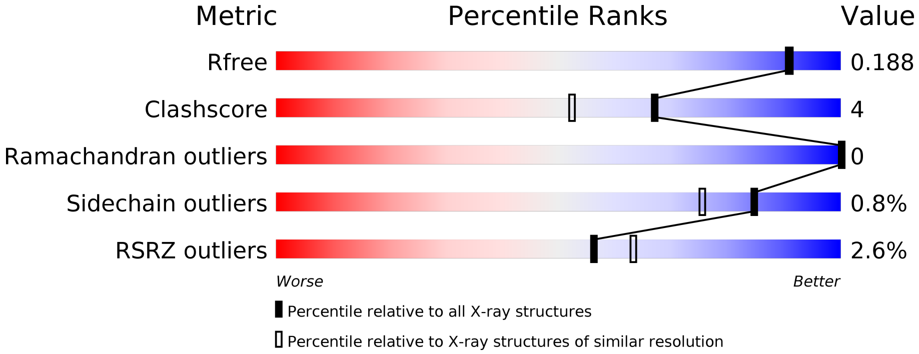

Metric	Whole archive (#Entries)	Similar resolution (#Entries, resolution range(Å))
R_{free}	100719	1762 (1.76-1.76)
Clashscore	112137	1889 (1.76-1.76)
Ramachandran outliers	110173	1868 (1.76-1.76)
Sidechain outliers	110143	1868 (1.76-1.76)
RSRZ outliers	101464	1770 (1.76-1.76)

The table below summarises the geometric issues observed across the polymeric chains and their fit to the electron density. The red, orange, yellow and green segments on the lower bar indicate the fraction of residues that contain outliers for >=3, 2, 1 and 0 types of geometric quality criteria. A grey segment represents the fraction of residues that are not modelled. The numeric value for each fraction is indicated below the corresponding segment, with a dot representing fractions <=5% The upper red bar (where present) indicates the fraction of residues that have poor fit to the electron density. The numeric value is given above the bar.

Mol	Chain	Length	Quality of chain
1	A	323	2% / 89% 6% 5%

The following table lists non-polymeric compounds, carbohydrate monomers and non-standard residues in protein, DNA, RNA chains that are outliers for geometric or electron-density-fit criteria:

Mol	Type	Chain	Res	Chirality	Geometry	Clashes	Electron density
3	ADP	A	402	-	-	-	X
5	NA	A	404	-	-	-	X

2 Entry composition (i)

There are 6 unique types of molecules in this entry. The entry contains 5036 atoms, of which 2297 are hydrogens and 0 are deuteriums.

In the tables below, the ZeroOcc column contains the number of atoms modelled with zero occupancy, the AltConf column contains the number of residues with at least one atom in alternate conformation and the Trace column contains the number of residues modelled with at most 2 atoms.

- Molecule 1 is a protein called Fructokinase.

Mol	Chain	Residues	Atoms						ZeroOcc	AltConf	Trace
			Total	C	H	N	O	S			
1	A	308	4621	1485	2273	402	451	10	0	2	0

- Molecule 2 is FRUCTOSE (three-letter code: FRU) (formula: $C_6H_{12}O_6$).

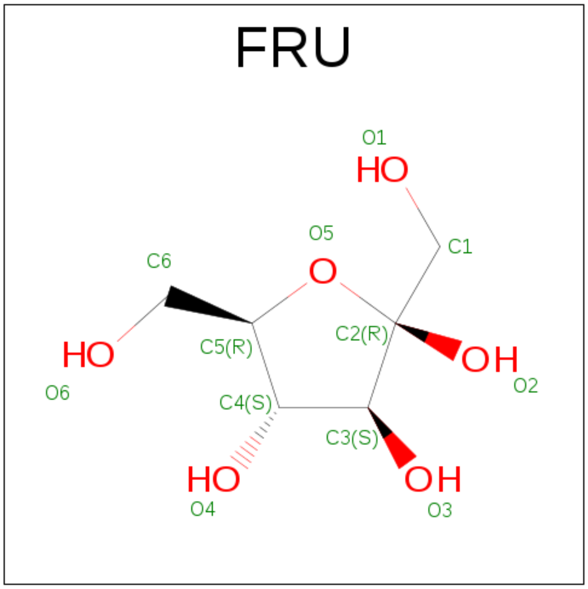

Mol	Chain	Residues	Atoms				ZeroOcc	AltConf
			Total	C	H	O		
2	A	1	24	6	12	6	0	0

- Molecule 3 is ADENOSINE-5'-DIPHOSPHATE (three-letter code: ADP) (formula: $C_{10}H_{15}N_5O_{10}P_2$).

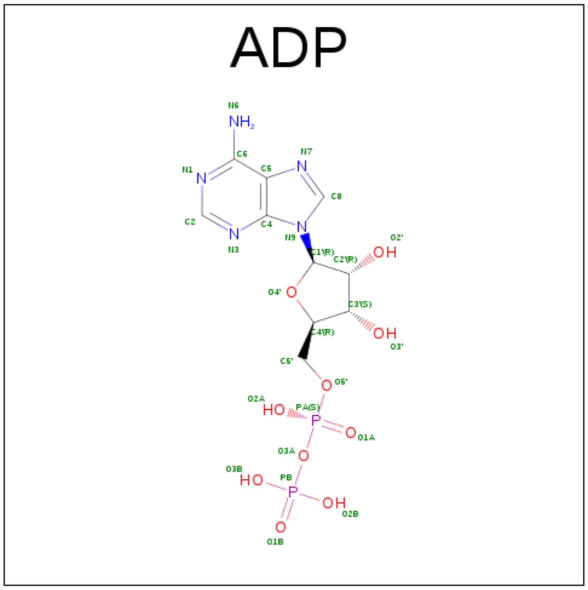

Mol	Chain	Residues	Atoms						ZeroOcc	AltConf
			Total	C	H	N	O	P		
3	A	1	39	10	12	5	10	2	0	0

- Molecule 4 is CALCIUM ION (three-letter code: CA) (formula: Ca).

Mol	Chain	Residues	Atoms		ZeroOcc	AltConf
			Total	Ca		
4	A	1	1	1	0	0

- Molecule 5 is SODIUM ION (three-letter code: NA) (formula: Na).

Mol	Chain	Residues	Atoms		ZeroOcc	AltConf
			Total	Na		
5	A	1	1	1	0	0

- Molecule 6 is water.

Mol	Chain	Residues	Atoms		ZeroOcc	AltConf
			Total	O		
6	A	350	350	350	0	0

3 Residue-property plots (i)

These plots are drawn for all protein, RNA and DNA chains in the entry. The first graphic for a chain summarises the proportions of the various outlier classes displayed in the second graphic. The second graphic shows the sequence view annotated by issues in geometry and electron density. Residues are color-coded according to the number of geometric quality criteria for which they contain at least one outlier: green = 0, yellow = 1, orange = 2 and red = 3 or more. A red dot above a residue indicates a poor fit to the electron density (RSRZ > 2). Stretches of 2 or more consecutive residues without any outlier are shown as a green connector. Residues present in the sample, but not in the model, are shown in grey.

- Molecule 1: Fructokinase

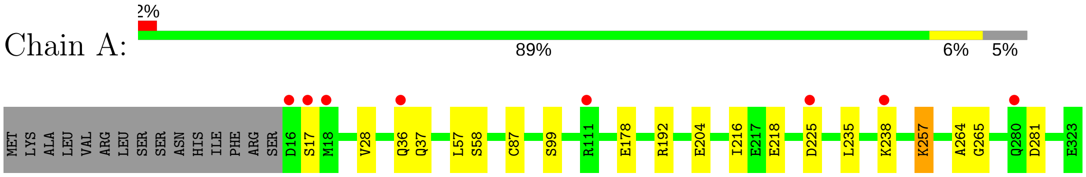

4 Data and refinement statistics (i)

Property	Value	Source
Space group	P 21 21 2	Depositor
Cell constants<br>a, b, c, α, β, γ	106.88Å 64.27Å 41.73Å<br>90.00° 90.00° 90.00°	Depositor
Resolution (Å)	35.00 – 1.75<br>38.87 – 1.75	Depositor<br>EDS
% Data completeness<br>(in resolution range)	99.0 (35.00-1.75)<br>99.1 (38.87-1.75)	Depositor<br>EDS
R_{merge}	0.04	Depositor
R_{sym}	(Not available)	Depositor
$< I/\sigma(I) >$ [1]	2.45 (at 1.75Å)	Xtriage
Refinement program	PHENIX (phenix.refine: 1.8_1069)	Depositor
R, R_{free}	0.148 , 0.186<br>0.148 , 0.188	Depositor<br>DCC
R_{free} test set	1497 reflections (5.08%)	DCC
Wilson B-factor (Å^2)	16.8	Xtriage
Anisotropy	0.271	Xtriage
Bulk solvent k_{sol}(e/Å^3), B_{sol}(Å^2)	0.43 , 49.5	EDS
L-test for twinning[2]	$< \|L\| > = 0.50$, $< L^2 > = 0.34$	Xtriage
Estimated twinning fraction	No twinning to report.	Xtriage
F_o,F_c correlation	0.96	EDS
Total number of atoms	5036	wwPDB-VP
Average B, all atoms (Å^2)	17.0	wwPDB-VP

Xtriage's analysis on translational NCS is as follows: *The largest off-origin peak in the Patterson function is 6.41% of the height of the origin peak. No significant pseudotranslation is detected.*

[1]Intensities estimated from amplitudes.

[2]Theoretical values of $< |L| >$, $< L^2 >$ for acentric reflections are 0.5, 0.333 respectively for untwinned datasets, and 0.375, 0.2 for perfectly twinned datasets.

5 Model quality (i)

5.1 Standard geometry (i)

Bond lengths and bond angles in the following residue types are not validated in this section: NA, CA, FRU, ADP

The Z score for a bond length (or angle) is the number of standard deviations the observed value is removed from the expected value. A bond length (or angle) with $|Z| > 5$ is considered an outlier worth inspection. RMSZ is the root-mean-square of all Z scores of the bond lengths (or angles).

Mol	Chain	Bond lengths		Bond angles	
		RMSZ	$\#\|Z\| >5$	RMSZ	$\#\|Z\| >5$
1	A	0.51	1/2404 (0.0%)	0.62	0/3271

All (1) bond length outliers are listed below:

Mol	Chain	Res	Type	Atoms	Z	Observed(Å)	Ideal(Å)
1	A	87	CYS	CB-SG	-5.47	1.73	1.81

There are no bond angle outliers.

There are no chirality outliers.

There are no planarity outliers.

5.2 Too-close contacts (i)

In the following table, the Non-H and H(model) columns list the number of non-hydrogen atoms and hydrogen atoms in the chain respectively. The H(added) column lists the number of hydrogen atoms added and optimized by MolProbity. The Clashes column lists the number of clashes within the asymmetric unit, whereas Symm-Clashes lists symmetry related clashes.

Mol	Chain	Non-H	H(model)	H(added)	Clashes	Symm-Clashes
1	A	2348	2273	2300	16	0
2	A	12	12	12	0	0
3	A	27	12	12	8	0
4	A	1	0	0	0	0
5	A	1	0	0	0	0
6	A	350	0	0	8	4
All	All	2739	2297	2324	21	4

The all-atom clashscore is defined as the number of clashes found per 1000 atoms (including hydrogen atoms). The all-atom clashscore for this structure is 4.

All (21) close contacts within the same asymmetric unit are listed below, sorted by their clash magnitude.

Atom-1	Atom-2	Interatomic distance (Å)	Clash overlap (Å)
3:A:402:ADP:O3B	6:A:501:HOH:O	1.85	0.94
1:A:178:GLU:OE2	6:A:502:HOH:O	2.00	0.79
1:A:257:LYS:HD3	1:A:257:LYS:O	1.83	0.77
1:A:257:LYS:CD	1:A:257:LYS:O	2.41	0.68
1:A:264:ALA:HB3	3:A:402:ADP:O3A	1.94	0.68
3:A:402:ADP:O5'	3:A:402:ADP:H8	1.88	0.57
1:A:28:VAL:HG22	1:A:99:SER:HB3	1.88	0.56
1:A:265:GLY:H	3:A:402:ADP:PB	2.33	0.51
1:A:218:GLU:OE2	6:A:503:HOH:O	2.19	0.50
3:A:402:ADP:O1B	6:A:504:HOH:O	2.20	0.48
1:A:204:GLU:HG2	1:A:216:ILE:HD11	1.95	0.48
1:A:235:LEU:HB3	1:A:238:LYS:HE3	1.96	0.48
1:A:257:LYS:CG	1:A:257:LYS:O	2.61	0.48
1:A:281:ASP:CG	6:A:507:HOH:O	2.53	0.47
1:A:57:LEU:O	1:A:58[B]:SER:HB2	2.15	0.45
3:A:402:ADP:O2A	6:A:505:HOH:O	2.21	0.44
1:A:265:GLY:HA3	3:A:402:ADP:O3B	2.18	0.43
1:A:192:ARG:NH2	6:A:510:HOH:O	2.32	0.42
1:A:225:ASP:N	1:A:225:ASP:OD1	2.54	0.41
1:A:36:GLN:O	1:A:37:GLN:HB2	2.21	0.40
3:A:402:ADP:N7	6:A:515:HOH:O	2.51	0.40

All (4) symmetry-related close contacts are listed below. The label for Atom-2 includes the symmetry operator and encoded unit-cell translations to be applied.

Atom-1	Atom-2	Interatomic distance (Å)	Clash overlap (Å)
6:A:807:HOH:O	6:A:839:HOH:O[2_565]	1.85	0.35
6:A:538:HOH:O	6:A:811:HOH:O[1_554]	2.06	0.14
6:A:736:HOH:O	6:A:760:HOH:O[1_554]	2.09	0.11
6:A:819:HOH:O	6:A:839:HOH:O[2_565]	2.17	0.03

5.3 Torsion angles (i)

5.3.1 Protein backbone (i)

In the following table, the Percentiles column shows the percent Ramachandran outliers of the chain as a percentile score with respect to all X-ray entries followed by that with respect to entries of similar resolution.

The Analysed column shows the number of residues for which the backbone conformation was analysed, and the total number of residues.

Mol	Chain	Analysed	Favoured	Allowed	Outliers	Percentiles	
1	A	308/323 (95%)	304 (99%)	4 (1%)	0	100	100

There are no Ramachandran outliers to report.

5.3.2 Protein sidechains (i)

In the following table, the Percentiles column shows the percent sidechain outliers of the chain as a percentile score with respect to all X-ray entries followed by that with respect to entries of similar resolution.

The Analysed column shows the number of residues for which the sidechain conformation was analysed, and the total number of residues.

Mol	Chain	Analysed	Rotameric	Outliers	Percentiles	
1	A	250/262 (95%)	248 (99%)	2 (1%)	85	75

All (2) residues with a non-rotameric sidechain are listed below:

Mol	Chain	Res	Type
1	A	17	SER
1	A	257	LYS

Some sidechains can be flipped to improve hydrogen bonding and reduce clashes. All (2) such sidechains are listed below:

Mol	Chain	Res	Type
1	A	91	HIS
1	A	227	GLN

5.3.3 RNA (i)

There are no RNA molecules in this entry.

5.4 Non-standard residues in protein, DNA, RNA chains (i)

There are no non-standard protein/DNA/RNA residues in this entry.

5.5 Carbohydrates (i)

There are no carbohydrates in this entry.

5.6 Ligand geometry (i)

Of 4 ligands modelled in this entry, 2 are monoatomic - leaving 2 for Mogul analysis.

In the following table, the Counts columns list the number of bonds (or angles) for which Mogul statistics could be retrieved, the number of bonds (or angles) that are observed in the model and the number of bonds (or angles) that are defined in the chemical component dictionary. The Link column lists molecule types, if any, to which the group is linked. The Z score for a bond length (or angle) is the number of standard deviations the observed value is removed from the expected value. A bond length (or angle) with $|Z| > 2$ is considered an outlier worth inspection. RMSZ is the root-mean-square of all Z scores of the bond lengths (or angles).

Mol	Type	Chain	Res	Link	Bond lengths			Bond angles		
					Counts	RMSZ	$\#\|Z\|>2$	Counts	RMSZ	$\#\|Z\|>2$
2	FRU	A	401	-	11,12,12	1.25	1 (9%)	10,18,18	0.83	1 (10%)
3	ADP	A	402	-	25,29,29	1.45	5 (20%)	24,45,45	2.19	3 (12%)

In the following table, the Chirals column lists the number of chiral outliers, the number of chiral centers analysed, the number of these observed in the model and the number defined in the chemical component dictionary. Similar counts are reported in the Torsion and Rings columns. '-' means no outliers of that kind were identified.

Mol	Type	Chain	Res	Link	Chirals	Torsions	Rings
2	FRU	A	401	-	-	0/5/24/24	0/1/1/1
3	ADP	A	402	-	-	0/12/32/32	0/3/3/3

All (6) bond length outliers are listed below:

Mol	Chain	Res	Type	Atoms	Z	Observed(Å)	Ideal(Å)
3	A	402	ADP	C2'-C1'	-3.51	1.48	1.53
2	A	401	FRU	O5-C2	-2.58	1.39	1.43
3	A	402	ADP	C2'-C3'	-2.27	1.47	1.53
3	A	402	ADP	O2'-C2'	-2.02	1.38	1.43
3	A	402	ADP	O3'-C3'	-2.00	1.38	1.43
3	A	402	ADP	C6-N6	2.27	1.43	1.34

All (4) bond angle outliers are listed below:

Mol	Chain	Res	Type	Atoms	Z	Observed(^{o})	Ideal(^{o})
3	A	402	ADP	N3-C2-N1	-9.35	120.72	128.86

Continued on next page...

Continued from previous page...

Mol	Chain	Res	Type	Atoms	Z	Observed(°)	Ideal(°)
3	A	402	ADP	C4-C5-N7	-2.71	106.80	109.41
2	A	401	FRU	O2-C2-O5	-2.33	104.82	109.45
3	A	402	ADP	O5'-C5'-C4'	2.12	116.51	109.00

There are no chirality outliers.

There are no torsion outliers.

There are no ring outliers.

1 monomer is involved in 8 short contacts:

Mol	Chain	Res	Type	Clashes	Symm-Clashes
3	A	402	ADP	8	0

5.7 Other polymers (i)

There are no such residues in this entry.

5.8 Polymer linkage issues (i)

There are no chain breaks in this entry.

6 Fit of model and data (i)

6.1 Protein, DNA and RNA chains (i)

In the following table, the column labelled '#RSRZ> 2' contains the number (and percentage) of RSRZ outliers, followed by percent RSRZ outliers for the chain as percentile scores relative to all X-ray entries and entries of similar resolution. The OWAB column contains the minimum, median, 95th percentile and maximum values of the occupancy-weighted average B-factor per residue. The column labelled 'Q< 0.9' lists the number of (and percentage) of residues with an average occupancy less than 0.9.

Mol	Chain	Analysed	<RSRZ>	#RSRZ>2			OWAB(Å^2)	Q<0.9
1	A	308/323 (95%)	-0.20	8 (2%)	56	63	5, 11, 32, 80	0

All (8) RSRZ outliers are listed below:

Mol	Chain	Res	Type	RSRZ
1	A	17	SER	7.8
1	A	16	ASP	7.8
1	A	36	GLN	4.1
1	A	280	GLN	2.6
1	A	18	MET	2.5
1	A	111	ARG	2.3
1	A	225	ASP	2.1
1	A	238	LYS	2.0

6.2 Non-standard residues in protein, DNA, RNA chains (i)

There are no non-standard protein/DNA/RNA residues in this entry.

6.3 Carbohydrates (i)

There are no carbohydrates in this entry.

6.4 Ligands (i)

In the following table, the Atoms column lists the number of modelled atoms in the group and the number defined in the chemical component dictionary. LLDF column lists the quality of electron density of the group with respect to its neighbouring residues in protein, DNA or RNA chains. The B-factors column lists the minimum, median, 95th percentile and maximum values of B factors of atoms in the group. The column labelled 'Q< 0.9' lists the number of atoms with occupancy less than 0.9.

Mol	Type	Chain	Res	Atoms	RSCC	RSR	LLDF	B-factors($Å^2$)	Q<0.9
5	NA	A	404	1/1	0.84	0.31	17.99	35,35,35,35	0
3	ADP	A	402	27/27	0.70	0.33	6.36	31,51,63,128	0
4	CA	A	403	1/1	1.00	0.10	1.43	19,19,19,19	0
2	FRU	A	401	12/12	0.99	0.05	-1.11	3,6,8,8	0

6.5 Other polymers (i)

There are no such residues in this entry.

CONFIDENTIAL VALIDATION REPORT

WORLDWIDE PDB PROTEIN DATA BANK

Full wwPDB X-ray Structure Validation Report (i)

Sep 26, 2017 – 03:33 PM JST

PDB ID	:	5YGG
Title	:	Crystal structure of Fructokinase Double-Mutant (T261C-H108C) from Vibrio cholerae O395 in fructose, ADP and potassium ion bound form
Deposited on	:	2017-09-22
Resolution	:	1.67 Å(reported)

This is a Full wwPDB X-ray Structure Validation Report.

This report is produced by the wwPDB biocuration pipeline after annotation of the structure.

We welcome your comments at *validation@mail.wwpdb.org*
A user guide is available at
http://wwpdb.org/validation/2016/XrayValidationReportHelp
with specific help available everywhere you see the (i) symbol.

The following versions of software and data (see references (i)) were used in the production of this report:

MolProbity	:	4.02b-467
Mogul	:	1.7.2 (RC1), CSD as538be (2017)
Xtriage (Phenix)	:	1.9-1692
EDS	:	rb-20030345
Percentile statistics	:	20161228.v01 (using entries in the PDB archive December 28th 2016)
Refmac	:	5.8.0135
CCP4	:	6.5.0
Ideal geometry (proteins)	:	Engh & Huber (2001)
Ideal geometry (DNA, RNA)	:	Parkinson et al. (1996)
Validation Pipeline (wwPDB-VP)	:	rb-20030345

1 Overall quality at a glance (i)

The following experimental techniques were used to determine the structure:
X-RAY DIFFRACTION

The reported resolution of this entry is 1.67 Å.

Percentile scores (ranging between 0-100) for global validation metrics of the entry are shown in the following graphic. The table shows the number of entries on which the scores are based.

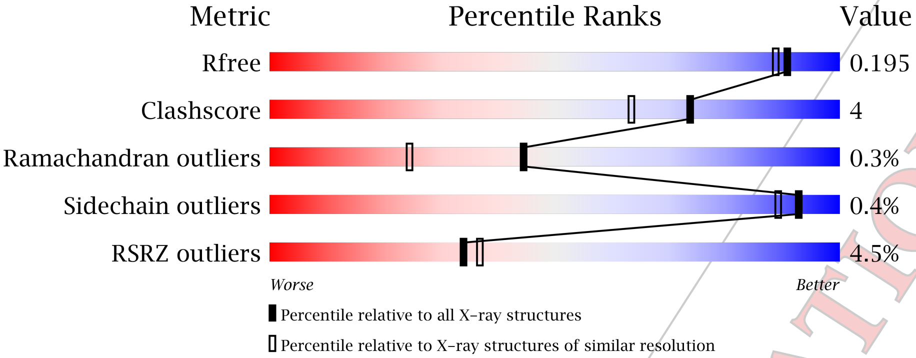

Metric	Whole archive (#Entries)	Similar resolution (#Entries, resolution range(Å))
R_{free}	100719	5252 (1.70-1.66)
Clashscore	112137	5803 (1.70-1.66)
Ramachandran outliers	110173	5704 (1.70-1.66)
Sidechain outliers	110143	5703 (1.70-1.66)
RSRZ outliers	101464	5298 (1.70-1.66)

The table below summarises the geometric issues observed across the polymeric chains and their fit to the electron density. The red, orange, yellow and green segments on the lower bar indicate the fraction of residues that contain outliers for >=3, 2, 1 and 0 types of geometric quality criteria. A grey segment represents the fraction of residues that are not modelled. The numeric value for each fraction is indicated below the corresponding segment, with a dot representing fractions <=5% The upper red bar (where present) indicates the fraction of residues that have poor fit to the electron density. The numeric value is given above the bar.

Mol	Chain	Length	Quality of chain
1	A	323	4% / 88% 7% •

The following table lists non-polymeric compounds, carbohydrate monomers and non-standard residues in protein, DNA, RNA chains that are outliers for geometric or electron-density-fit criteria:

Mol	Type	Chain	Res	Chirality	Geometry	Clashes	Electron density
2	ADP	A	601	-	-	-	X
4	CA	A	603	-	-	-	X

CONFIDENTIAL VALIDATION REPORT

2 Entry composition (i)

There are 6 unique types of molecules in this entry. The entry contains 2723 atoms, of which 0 are hydrogens and 0 are deuteriums.

In the tables below, the ZeroOcc column contains the number of atoms modelled with zero occupancy, the AltConf column contains the number of residues with at least one atom in alternate conformation and the Trace column contains the number of residues modelled with at most 2 atoms.

- Molecule 1 is a protein called Fructokinase.

Mol	Chain	Residues	Atoms					ZeroOcc	AltConf	Trace
			Total	C	N	O	S			
1	A	310	2340	1480	401	447	12	0	0	0

There are 2 discrepancies between the modelled and reference sequences:

Chain	Residue	Modelled	Actual	Comment	Reference
A	108	CYS	HIS	engineered mutation	UNP A0A0H3AER7
A	261	CYS	THR	engineered mutation	UNP A0A0H3AER7

- Molecule 2 is ADENOSINE-5'-DIPHOSPHATE (three-letter code: ADP) (formula: $C_{10}H_{15}N_5O_{10}P_2$).

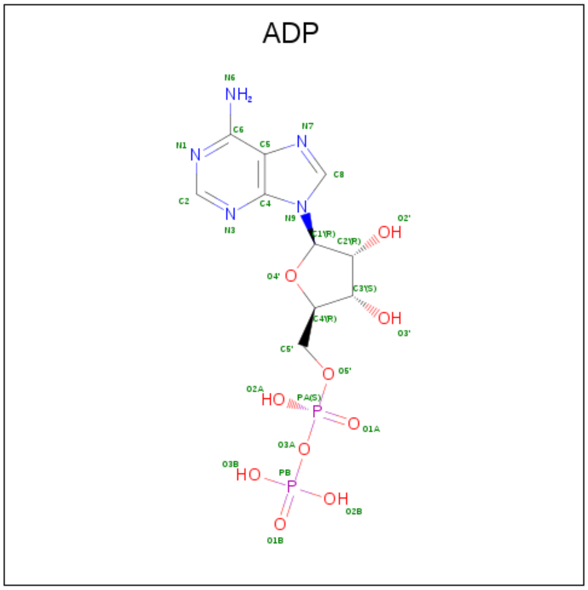

Mol	Chain	Residues	Atoms					ZeroOcc	AltConf
			Total	C	N	O	P		
2	A	1	27	10	5	10	2	0	0

- Molecule 3 is FRUCTOSE (three-letter code: FRU) (formula: $C_6H_{12}O_6$).

CONFIDENTIAL VALIDATION REPORT

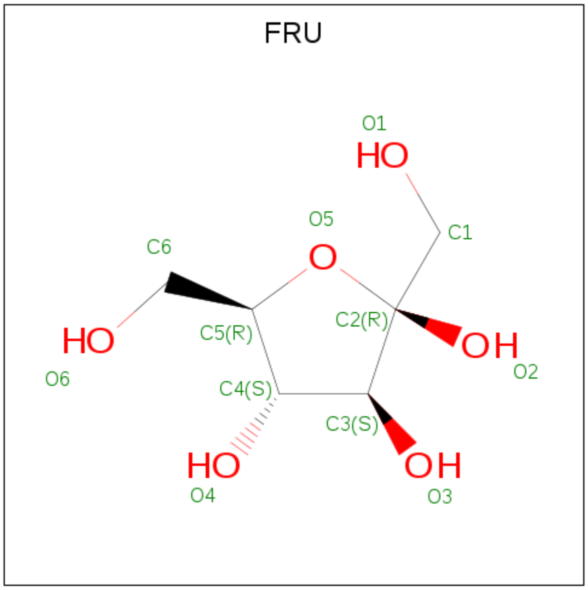

Mol	Chain	Residues	Atoms	ZeroOcc	AltConf
3	A	1	Total C O<br>12 6 6	0	0

- Molecule 4 is CALCIUM ION (three-letter code: CA) (formula: Ca).

Mol	Chain	Residues	Atoms	ZeroOcc	AltConf
4	A	1	Total Ca<br>1 1	0	0

- Molecule 5 is POTASSIUM ION (three-letter code: K) (formula: K).

Mol	Chain	Residues	Atoms	ZeroOcc	AltConf
5	A	1	Total K<br>1 1	0	0

- Molecule 6 is water.

Mol	Chain	Residues	Atoms	ZeroOcc	AltConf
6	A	342	Total O<br>342 342	0	0

CONFIDENTIAL VALIDATION REPORT

3 Residue-property plots (i)

These plots are drawn for all protein, RNA and DNA chains in the entry. The first graphic for a chain summarises the proportions of the various outlier classes displayed in the second graphic. The second graphic shows the sequence view annotated by issues in geometry and electron density. Residues are color-coded according to the number of geometric quality criteria for which they contain at least one outlier: green = 0, yellow = 1, orange = 2 and red = 3 or more. A red dot above a residue indicates a poor fit to the electron density (RSRZ > 2). Stretches of 2 or more consecutive residues without any outlier are shown as a green connector. Residues present in the sample, but not in the model, are shown in grey.

- Molecule 1: Fructokinase

Chain A: 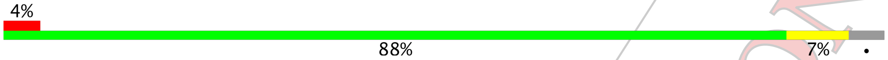

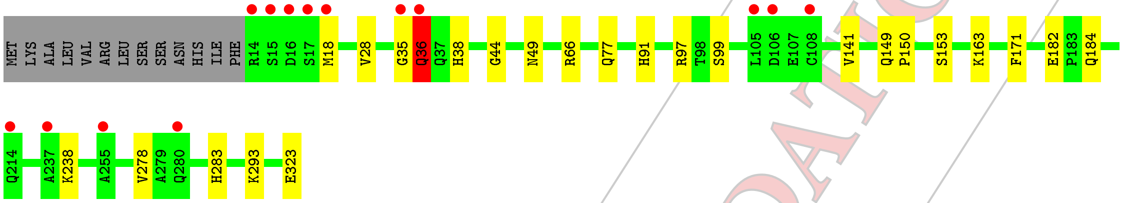

CONFIDENTIAL VALIDATION REPORT

4 Data and refinement statistics (i)

Property	Value	Source
Space group	P 2 21 21	Depositor
Cell constants<br>a, b, c, α, β, γ	41.76Å 64.33Å 116.40Å<br>90.00° 90.00° 90.00°	Depositor
Resolution (Å)	10.06 – 1.67<br>35.03 – 1.67	Depositor<br>EDS
% Data completeness<br>(in resolution range)	98.4 (10.06-1.67)<br>94.7 (35.03-1.67)	Depositor<br>EDS
R_{merge}	(Not available)	Depositor
R_{sym}	(Not available)	Depositor
$< I/\sigma(I) >$ [1]	1.74 (at 1.67Å)	Xtriage
Refinement program	PHENIX 1.9_1692	Depositor
R, R_{free}	0.163 , 0.195<br>0.163 , 0.195	Depositor<br>DCC
R_{free} test set	1775 reflections (4.86%)	DCC
Wilson B-factor (Å^2)	12.6	Xtriage
Anisotropy	0.446	Xtriage
Bulk solvent k_{sol}(e/Å^3), B_{sol}(Å^2)	0.36 , 52.0	EDS
L-test for twinning[2]	$< \|L\| >$ = 0.47, $< L^2 >$ = 0.30	Xtriage
Estimated twinning fraction	No twinning to report.	Xtriage
F_o,F_c correlation	0.96	EDS
Total number of atoms	2723	wwPDB-VP
Average B, all atoms (Å^2)	18.0	wwPDB-VP

Xtriage's analysis on translational NCS is as follows: *The largest off-origin peak in the Patterson function is 9.09% of the height of the origin peak. No significant pseudotranslation is detected.*

[1]Intensities estimated from amplitudes.

[2]Theoretical values of $< |L| >$, $< L^2 >$ for acentric reflections are 0.5, 0.333 respectively for untwinned datasets, and 0.375, 0.2 for perfectly twinned datasets.

CONFIDENTIAL VALIDATION REPORT

5 Model quality (i)

5.1 Standard geometry (i)

Bond lengths and bond angles in the following residue types are not validated in this section: K, FRU, CA, ADP

The Z score for a bond length (or angle) is the number of standard deviations the observed value is removed from the expected value. A bond length (or angle) with $|Z| > 5$ is considered an outlier worth inspection. RMSZ is the root-mean-square of all Z scores of the bond lengths (or angles).

Mol	Chain	Bond lengths		Bond angles	
		RMSZ	$\#\|Z\| > 5$	RMSZ	$\#\|Z\| > 5$
1	A	0.36	0/2389	0.53	0/3251

Chiral center outliers are detected by calculating the chiral volume of a chiral center and verifying if the center is modelled as a planar moiety or with the opposite hand.A planarity outlier is detected by checking planarity of atoms in a peptide group, atoms in a mainchain group or atoms of a sidechain that are expected to be planar.

Mol	Chain	#Chirality outliers	#Planarity outliers
1	A	0	2

There are no bond length outliers.

There are no bond angle outliers.

There are no chirality outliers.

All (2) planarity outliers are listed below:

Mol	Chain	Res	Type	Group
1	A	35	GLY	Peptide
1	A	36	GLN	Peptide

5.2 Too-close contacts (i)

In the following table, the Non-H and H(model) columns list the number of non-hydrogen atoms and hydrogen atoms in the chain respectively. The H(added) column lists the number of hydrogen atoms added and optimized by MolProbity. The Clashes column lists the number of clashes within the asymmetric unit, whereas Symm-Clashes lists symmetry related clashes.

Mol	Chain	Non-H	H(model)	H(added)	Clashes	Symm-Clashes
1	A	2340	0	2280	18	0

Continued on next page...

CONFIDENTIAL VALIDATION REPORT

Continued from previous page...

Mol	Chain	Non-H	H(model)	H(added)	Clashes	Symm-Clashes
2	A	27	0	12	0	0
3	A	12	0	11	0	0
4	A	1	0	0	0	0
5	A	1	0	0	0	0
6	A	342	0	0	8	6
All	All	2723	0	2303	18	6

The all-atom clashscore is defined as the number of clashes found per 1000 atoms (including hydrogen atoms). The all-atom clashscore for this structure is 4.

All (18) close contacts within the same asymmetric unit are listed below, sorted by their clash magnitude.

Atom-1	Atom-2	Interatomic distance (Å)	Clash overlap (Å)
1:A:36:GLN:HA	1:A:38:HIS:H	1.43	0.83
1:A:323:GLU:OE2	6:A:701:HOH:O	2.00	0.80
1:A:44:GLY:HA2	1:A:49:ASN:HD21	1.65	0.61
1:A:77:GLN:NE2	6:A:702:HOH:O	2.26	0.59
1:A:149:GLN:HE22	1:A:153:SER:H	1.56	0.54
1:A:97:ARG:NH2	6:A:708:HOH:O	2.40	0.54
1:A:283:HIS:HE1	6:A:795:HOH:O	1.93	0.51
1:A:91:HIS:HE1	6:A:823:HOH:O	1.96	0.48
1:A:141:VAL:HG23	1:A:171:PHE:HD1	1.78	0.48
1:A:238:LYS:HE3	1:A:238:LYS:HB3	1.52	0.45
1:A:163:LYS:NZ	6:A:705:HOH:O	2.31	0.44
1:A:293:LYS:HE3	6:A:1009:HOH:O	2.18	0.44
1:A:18:MET:HG3	1:A:278:VAL:HG12	2.00	0.43
1:A:149:GLN:HE21	1:A:150:PRO:HA	1.83	0.42
1:A:293:LYS:HG3	6:A:1009:HOH:O	2.21	0.41
1:A:28:VAL:HG12	1:A:99:SER:HB3	2.02	0.41
1:A:141:VAL:HG23	1:A:171:PHE:CD1	2.55	0.41
1:A:182:GLU:HB3	1:A:184:GLN:NE2	2.36	0.41

All (6) symmetry-related close contacts are listed below. The label for Atom-2 includes the symmetry operator and encoded unit-cell translations to be applied.

Atom-1	Atom-2	Interatomic distance (Å)	Clash overlap (Å)
6:A:746:HOH:O	6:A:878:HOH:O[3_557]	1.88	0.32
6:A:732:HOH:O	6:A:886:HOH:O[1_655]	2.03	0.17
6:A:931:HOH:O	6:A:990:HOH:O[2_567]	2.12	0.08
6:A:894:HOH:O	6:A:1001:HOH:O[3_657]	2.13	0.07

Continued on next page...

CONFIDENTIAL VALIDATION REPORT

Continued from previous page...

Atom-1	Atom-2	Interatomic distance (Å)	Clash overlap (Å)
6:A:900:HOH:O	6:A:958:HOH:O[3_557]	2.14	0.06
6:A:1010:HOH:O	6:A:1031:HOH:O[1_455]	2.18	0.02

5.3 Torsion angles (i)

5.3.1 Protein backbone (i)

In the following table, the Percentiles column shows the percent Ramachandran outliers of the chain as a percentile score with respect to all X-ray entries followed by that with respect to entries of similar resolution.

The Analysed column shows the number of residues for which the backbone conformation was analysed, and the total number of residues.

Mol	Chain	Analysed	Favoured	Allowed	Outliers	Percentiles	
1	A	308/323 (95%)	303 (98%)	4 (1%)	1 (0%)	44	24

All (1) Ramachandran outliers are listed below:

Mol	Chain	Res	Type
1	A	36	GLN

5.3.2 Protein sidechains (i)

In the following table, the Percentiles column shows the percent sidechain outliers of the chain as a percentile score with respect to all X-ray entries followed by that with respect to entries of similar resolution.

The Analysed column shows the number of residues for which the sidechain conformation was analysed, and the total number of residues.

Mol	Chain	Analysed	Rotameric	Outliers	Percentiles	
1	A	246/262 (94%)	245 (100%)	1 (0%)	93	89

All (1) residues with a non-rotameric sidechain are listed below:

Mol	Chain	Res	Type
1	A	66	ARG

Some sidechains can be flipped to improve hydrogen bonding and reduce clashes. All (7) such

CONFIDENTIAL VALIDATION REPORT

sidechains are listed below:

Mol	Chain	Res	Type
1	A	49	ASN
1	A	91	HIS
1	A	96	HIS
1	A	149	GLN
1	A	227	GLN
1	A	283	HIS
1	A	315	GLN

5.3.3 RNA (i)

There are no RNA molecules in this entry.

5.4 Non-standard residues in protein, DNA, RNA chains (i)

There are no non-standard protein/DNA/RNA residues in this entry.

5.5 Carbohydrates (i)

There are no carbohydrates in this entry.

5.6 Ligand geometry (i)

Of 4 ligands modelled in this entry, 2 are monoatomic - leaving 2 for Mogul analysis.

In the following table, the Counts columns list the number of bonds (or angles) for which Mogul statistics could be retrieved, the number of bonds (or angles) that are observed in the model and the number of bonds (or angles) that are defined in the Chemical Component Dictionary. The Link column lists molecule types, if any, to which the group is linked. The Z score for a bond length (or angle) is the number of standard deviations the observed value is removed from the expected value. A bond length (or angle) with $|Z| > 2$ is considered an outlier worth inspection. RMSZ is the root-mean-square of all Z scores of the bond lengths (or angles).

Mol	Type	Chain	Res	Link	Bond lengths			Bond angles		
					Counts	RMSZ	$\#\|Z\| > 2$	Counts	RMSZ	$\#\|Z\| > 2$
2	ADP	A	601	4	25,29,29	3.25	9 (36%)	24,45,45	3.33	5 (20%)
3	FRU	A	602	-	11,12,12	4.24	6 (54%)	10,18,18	0.71	0

In the following table, the Chirals column lists the number of chiral outliers, the number of chiral centers analysed, the number of these observed in the model and the number defined in the Chemical Component Dictionary. Similar counts are reported in the Torsion and Rings columns.

CONFIDENTIAL VALIDATION REPORT

'-' means no outliers of that kind were identified.

Mol	Type	Chain	Res	Link	Chirals	Torsions	Rings
2	ADP	A	601	4	-	0/12/32/32	0/3/3/3
3	FRU	A	602	-	-	0/5/24/24	0/1/1/1

All (15) bond length outliers are listed below:

Mol	Chain	Res	Type	Atoms	Z	Observed(Å)	Ideal(Å)
2	A	601	ADP	C2'-C3'	-9.61	1.28	1.53
2	A	601	ADP	O4'-C1'	-7.19	1.31	1.41
3	A	602	FRU	O2-C2	-6.70	1.29	1.40
3	A	602	FRU	O5-C5	-5.58	1.31	1.43
2	A	601	ADP	C5'-C4'	-4.71	1.36	1.51
3	A	602	FRU	C4-C3	-3.99	1.34	1.52
2	A	601	ADP	O2'-C2'	2.01	1.47	1.43
2	A	601	ADP	C4-N3	2.15	1.38	1.35
2	A	601	ADP	O3'-C3'	2.23	1.48	1.43
2	A	601	ADP	C2-N3	2.41	1.36	1.32
3	A	602	FRU	O3-C3	2.85	1.48	1.42
2	A	601	ADP	O4'-C4'	3.55	1.53	1.45
3	A	602	FRU	C4-C5	3.95	1.63	1.53
2	A	601	ADP	C2'-C1'	6.85	1.64	1.53
3	A	602	FRU	O5-C2	8.76	1.56	1.43

All (5) bond angle outliers are listed below:

Mol	Chain	Res	Type	Atoms	Z	Observed(^{o})	Ideal(^{o})
2	A	601	ADP	N3-C2-N1	-9.25	120.80	128.86
2	A	601	ADP	N6-C6-N1	-8.00	102.91	118.77
2	A	601	ADP	C1'-N9-C4	-6.21	115.90	126.64
2	A	601	ADP	C4'-O4'-C1'	-2.43	107.19	109.77
2	A	601	ADP	C5-C6-N6	8.18	137.13	120.47

There are no chirality outliers.

There are no torsion outliers.

There are no ring outliers.

No monomer is involved in short contacts.

5.7 Other polymers (i)

There are no such residues in this entry.

CONFIDENTIAL VALIDATION REPORT

5.8 Polymer linkage issues (i)

There are no chain breaks in this entry.

CONFIDENTIAL VALIDATION REPORT

6 Fit of model and data (i)

6.1 Protein, DNA and RNA chains (i)

In the following table, the column labelled '#RSRZ> 2' contains the number (and percentage) of RSRZ outliers, followed by percent RSRZ outliers for the chain as percentile scores relative to all X-ray entries and entries of similar resolution. The OWAB column contains the minimum, median, 95th percentile and maximum values of the occupancy-weighted average B-factor per residue. The column labelled 'Q< 0.9' lists the number of (and percentage) of residues with an average occupancy less than 0.9.

Mol	Chain	Analysed	<RSRZ>	#RSRZ>2			OWAB(Å^2)	Q<0.9
1	A	310/323 (95%)	0.19	14 (4%)	34	37	7, 13, 34, 85	0

All (14) RSRZ outliers are listed below:

Mol	Chain	Res	Type	RSRZ
1	A	14	ARG	13.8
1	A	15	SER	13.6
1	A	17	SER	9.5
1	A	16	ASP	9.4
1	A	36	GLN	5.9
1	A	280	GLN	3.3
1	A	214	GLN	2.9
1	A	255	ALA	2.7
1	A	108	CYS	2.7
1	A	18	MET	2.7
1	A	105	LEU	2.5
1	A	35	GLY	2.5
1	A	237	ALA	2.4
1	A	106	ASP	2.1

6.2 Non-standard residues in protein, DNA, RNA chains (i)

There are no non-standard protein/DNA/RNA residues in this entry.

6.3 Carbohydrates (i)

There are no carbohydrates in this entry.

CONFIDENTIAL VALIDATION REPORT

6.4 Ligands (i)

In the following table, the Atoms column lists the number of modelled atoms in the group and the number defined in the chemical component dictionary. LLDF column lists the quality of electron density of the group with respect to its neighbouring residues in protein, DNA or RNA chains. The B-factors column lists the minimum, median, 95th percentile and maximum values of B factors of atoms in the group. The column labelled 'Q< 0.9' lists the number of atoms with occupancy less than 0.9.

Mol	Type	Chain	Res	Atoms	RSCC	RSR	LLDF	B-factors(Å^2)	Q<0.9
4	CA	A	603	1/1	0.90	0.29	13.64	78,78,78,78	0
2	ADP	A	601	27/27	0.61	0.28	3.25	36,49,116,161	0
3	FRU	A	602	12/12	0.93	0.11	0.82	9,14,17,18	0
5	K	A	604	1/1	0.99	0.05	-2.23	12,12,12,12	0

6.5 Other polymers (i)

There are no such residues in this entry.

CONFIDENTIAL VALIDATION REPORT

Full wwPDB X-ray Structure Validation Report (i)

Feb 13, 2017 – 12:58 am GMT

PDB ID	:	5F11
Title	:	Crystal structure of Fructokinase from Vibrio cholerae O395 in fructose bound form
Authors	:	Paul, R.; Nath, S.; Sen, U.
Deposited on	:	2015-11-28
Resolution	:	2.30 Å(reported)

This is a Full wwPDB X-ray Structure Validation Report for a publicly released PDB entry.

We welcome your comments at *validation@mail.wwpdb.org*
A user guide is available at
http://wwpdb.org/validation/2016/XrayValidationReportHelp
with specific help available everywhere you see the (i) symbol.

The following versions of software and data (see references (i)) were used in the production of this report:

MolProbity	:	4.02b-467
Mogul	:	1.7.2 (RC1), CSD as538be (2017)
Xtriage (Phenix)	:	1.9-1692
EDS	:	trunk28620
Percentile statistics	:	20161228.v01 (using entries in the PDB archive December 28th 2016)
Refmac	:	5.8.0135
CCP4	:	6.5.0
Ideal geometry (proteins)	:	Engh & Huber (2001)
Ideal geometry (DNA, RNA)	:	Parkinson et al. (1996)
Validation Pipeline (wwPDB-VP)	:	recalc28949

1 Overall quality at a glance (i)

The following experimental techniques were used to determine the structure:
X-RAY DIFFRACTION

The reported resolution of this entry is 2.30 Å.

Percentile scores (ranging between 0-100) for global validation metrics of the entry are shown in the following graphic. The table shows the number of entries on which the scores are based.

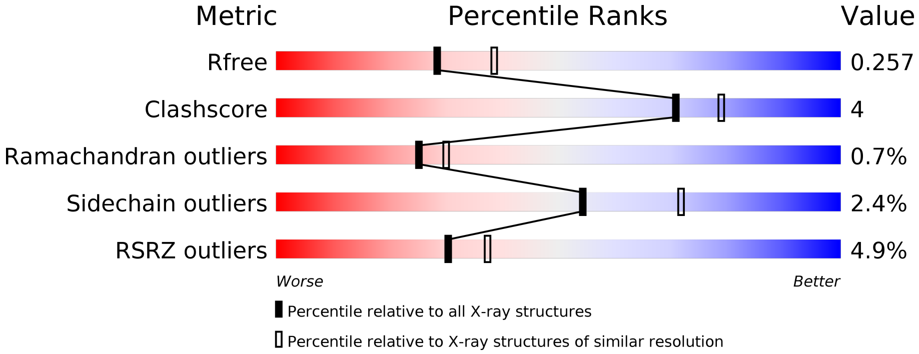

Metric	Whole archive (#Entries)	Similar resolution (#Entries, resolution range(Å))
R_{free}	100719	4130 (2.30-2.30)
Clashscore	112137	4751 (2.30-2.30)
Ramachandran outliers	110173	4705 (2.30-2.30)
Sidechain outliers	110143	4704 (2.30-2.30)
RSRZ outliers	101464	4156 (2.30-2.30)

The table below summarises the geometric issues observed across the polymeric chains and their fit to the electron density. The red, orange, yellow and green segments on the lower bar indicate the fraction of residues that contain outliers for >=3, 2, 1 and 0 types of geometric quality criteria. A grey segment represents the fraction of residues that are not modelled. The numeric value for each fraction is indicated below the corresponding segment, with a dot representing fractions <=5% The upper red bar (where present) indicates the fraction of residues that have poor fit to the electron density. The numeric value is given above the bar.

Mol	Chain	Length	Quality of chain
1	A	323	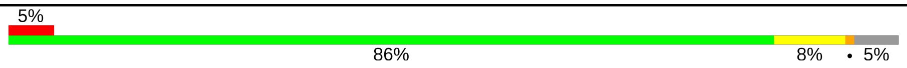

2 Entry composition (i)

There are 4 unique types of molecules in this entry. The entry contains 2555 atoms, of which 0 are hydrogens and 0 are deuteriums.

In the tables below, the ZeroOcc column contains the number of atoms modelled with zero occupancy, the AltConf column contains the number of residues with at least one atom in alternate conformation and the Trace column contains the number of residues modelled with at most 2 atoms.

- Molecule 1 is a protein called Fructokinase.

Mol	Chain	Residues	Atoms					ZeroOcc	AltConf	Trace
			Total	C	N	O	S			
1	A	308	2339	1479	401	449	10	0	0	0

- Molecule 2 is FRUCTOSE (three-letter code: FRU) (formula: $C_6H_{12}O_6$).

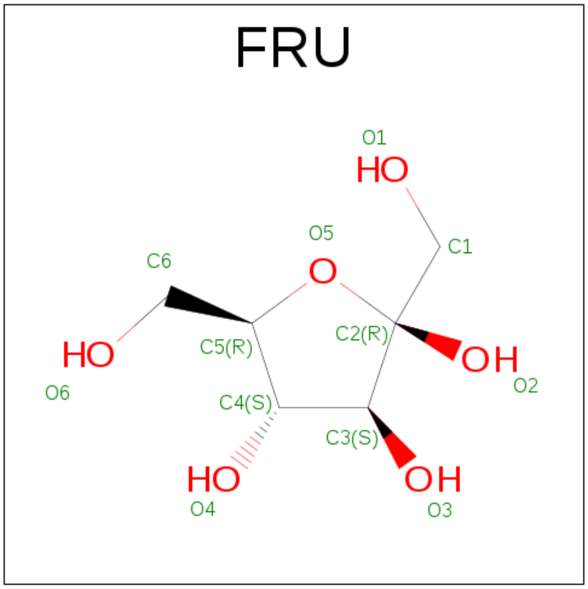

Mol	Chain	Residues	Atoms			ZeroOcc	AltConf
			Total	C	O		
2	A	1	12	6	6	0	0

- Molecule 3 is SODIUM ION (three-letter code: NA) (formula: Na).

Mol	Chain	Residues	Atoms		ZeroOcc	AltConf
			Total	Na		
3	A	1	1	1	0	0

- Molecule 4 is water.

Mol	Chain	Residues	Atoms		ZeroOcc	AltConf
4	A	203	Total<br>203	O<br>203	0	0

3 Residue-property plots (i)

These plots are drawn for all protein, RNA and DNA chains in the entry. The first graphic for a chain summarises the proportions of the various outlier classes displayed in the second graphic. The second graphic shows the sequence view annotated by issues in geometry and electron density. Residues are color-coded according to the number of geometric quality criteria for which they contain at least one outlier: green = 0, yellow = 1, orange = 2 and red = 3 or more. A red dot above a residue indicates a poor fit to the electron density (RSRZ > 2). Stretches of 2 or more consecutive residues without any outlier are shown as a green connector. Residues present in the sample, but not in the model, are shown in grey.

• Molecule 1: Fructokinase

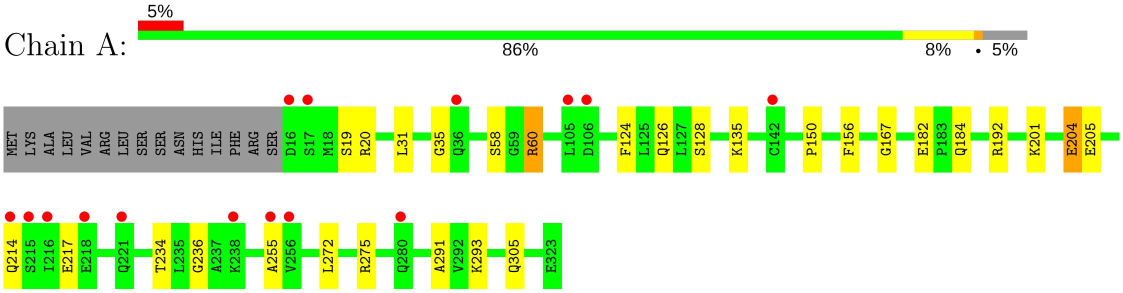

4 Data and refinement statistics (i)

Property	Value	Source
Space group	P 21 21 2	Depositor
Cell constants<br>a, b, c, α, β, γ	117.53Å 64.90Å 41.95Å<br>90.00° 90.00° 90.00°	Depositor
Resolution (Å)	25.67 – 2.30<br>26.20 – 2.30	Depositor<br>EDS
% Data completeness<br>(in resolution range)	95.6 (25.67-2.30)<br>95.7 (26.20-2.30)	Depositor<br>EDS
R_{merge}	0.10	Depositor
R_{sym}	(Not available)	Depositor
$< I/\sigma(I) >$ [1]	2.06 (at 2.31Å)	Xtriage
Refinement program	PHENIX (phenix.refine: 1.8_1069)	Depositor
R, R_{free}	0.187 , 0.258<br>0.188 , 0.257	Depositor<br>DCC
R_{free} test set	725 reflections (5.09%)	DCC
Wilson B-factor ($Å^2$)	27.8	Xtriage
Anisotropy	0.562	Xtriage
Bulk solvent k_{sol}(e/$Å^3$), B_{sol}($Å^2$)	0.34 , 59.6	EDS
L-test for twinning[2]	$< \|L\| > = 0.48$, $< L^2 > = 0.31$	Xtriage
Estimated twinning fraction	No twinning to report.	Xtriage
F_o,F_c correlation	0.94	EDS
Total number of atoms	2555	wwPDB-VP
Average B, all atoms ($Å^2$)	34.0	wwPDB-VP

Xtriage's analysis on translational NCS is as follows: *The largest off-origin peak in the Patterson function is 8.93% of the height of the origin peak. No significant pseudotranslation is detected.*

[1] Intensities estimated from amplitudes.

[2] Theoretical values of $< |L| >$, $< L^2 >$ for acentric reflections are 0.5, 0.333 respectively for untwinned datasets, and 0.375, 0.2 for perfectly twinned datasets.

5 Model quality (i)

5.1 Standard geometry (i)

Bond lengths and bond angles in the following residue types are not validated in this section: NA, FRU

The Z score for a bond length (or angle) is the number of standard deviations the observed value is removed from the expected value. A bond length (or angle) with $|Z| > 5$ is considered an outlier worth inspection. RMSZ is the root-mean-square of all Z scores of the bond lengths (or angles).

Mol	Chain	Bond lengths		Bond angles	
		RMSZ	#$|Z|$ >5	RMSZ	#$|Z|$ >5
1	A	0.38	0/2389	0.53	0/3251

There are no bond length outliers.

There are no bond angle outliers.

There are no chirality outliers.

There are no planarity outliers.

5.2 Too-close contacts (i)

In the following table, the Non-H and H(model) columns list the number of non-hydrogen atoms and hydrogen atoms in the chain respectively. The H(added) column lists the number of hydrogen atoms added and optimized by MolProbity. The Clashes column lists the number of clashes within the asymmetric unit, whereas Symm-Clashes lists symmetry related clashes.

Mol	Chain	Non-H	H(model)	H(added)	Clashes	Symm-Clashes
1	A	2339	0	2287	19	0
2	A	12	0	12	0	0
3	A	1	0	0	0	0
4	A	203	0	0	9	1
All	All	2555	0	2299	19	1

The all-atom clashscore is defined as the number of clashes found per 1000 atoms (including hydrogen atoms). The all-atom clashscore for this structure is 4.

All (19) close contacts within the same asymmetric unit are listed below, sorted by their clash magnitude.

Atom-1	Atom-2	Interatomic distance (Å)	Clash overlap (Å)
1:A:128:SER:OG	4:A:502:HOH:O	2.06	0.73
1:A:217:GLU:OE1	4:A:501:HOH:O	2.06	0.72
1:A:20:ARG:HB2	1:A:60:ARG:HH21	1.58	0.68
1:A:293:LYS:NZ	4:A:507:HOH:O	2.27	0.65
1:A:182:GLU:OE1	1:A:184:GLN:NE2	2.31	0.61
1:A:167:GLY:O	4:A:504:HOH:O	2.16	0.61
1:A:135:LYS:NZ	4:A:516:HOH:O	2.38	0.53
1:A:58:SER:OG	4:A:503:HOH:O	2.16	0.51
1:A:20:ARG:HB2	1:A:60:ARG:NH2	2.24	0.51
1:A:201:LYS:NZ	4:A:520:HOH:O	2.46	0.48
1:A:205:GLU:OE2	1:A:205:GLU:N	2.47	0.47
1:A:124:PHE:HA	1:A:150:PRO:HD2	1.98	0.45
1:A:156:PHE:CE1	1:A:192:ARG:HG2	2.53	0.43
1:A:272:LEU:HD12	1:A:291:ALA:HB1	2.01	0.43
1:A:305:GLN:NE2	4:A:515:HOH:O	2.37	0.43
1:A:182:GLU:HB3	1:A:184:GLN:NE2	2.34	0.42
1:A:255:ALA:N	4:A:506:HOH:O	2.26	0.41
1:A:275:ARG:HA	1:A:275:ARG:HD2	1.94	0.41
1:A:204:GLU:HG2	1:A:234:THR:O	2.22	0.40

All (1) symmetry-related close contacts are listed below. The label for Atom-2 includes the symmetry operator and encoded unit-cell translations to be applied.

Atom-1	Atom-2	Interatomic distance (Å)	Clash overlap (Å)
4:A:571:HOH:O	4:A:638:HOH:O[1_556]	2.03	0.17

5.3 Torsion angles (i)

5.3.1 Protein backbone (i)

In the following table, the Percentiles column shows the percent Ramachandran outliers of the chain as a percentile score with respect to all X-ray entries followed by that with respect to entries of similar resolution.

The Analysed column shows the number of residues for which the backbone conformation was analysed, and the total number of residues.

Mol	Chain	Analysed	Favoured	Allowed	Outliers	Percentiles
1	A	306/323 (95%)	297 (97%)	7 (2%)	2 (1%)	25 30

All (2) Ramachandran outliers are listed below:

Mol	Chain	Res	Type
1	A	236	GLY
1	A	35	GLY

5.3.2 Protein sidechains (i)

In the following table, the Percentiles column shows the percent sidechain outliers of the chain as a percentile score with respect to all X-ray entries followed by that with respect to entries of similar resolution.

The Analysed column shows the number of residues for which the sidechain conformation was analysed, and the total number of residues.

Mol	Chain	Analysed	Rotameric	Outliers	Percentiles	
1	A	248/262 (95%)	242 (98%)	6 (2%)	54	72

All (6) residues with a non-rotameric sidechain are listed below:

Mol	Chain	Res	Type
1	A	19	SER
1	A	31	LEU
1	A	60	ARG
1	A	126	GLN
1	A	204	GLU
1	A	214	GLN

Some sidechains can be flipped to improve hydrogen bonding and reduce clashes. There are no such sidechains identified.

5.3.3 RNA (i)

There are no RNA molecules in this entry.

5.4 Non-standard residues in protein, DNA, RNA chains (i)

There are no non-standard protein/DNA/RNA residues in this entry.

5.5 Carbohydrates (i)

There are no carbohydrates in this entry.

5.6 Ligand geometry (i)

Of 2 ligands modelled in this entry, 1 is monoatomic - leaving 1 for Mogul analysis.

In the following table, the Counts columns list the number of bonds (or angles) for which Mogul statistics could be retrieved, the number of bonds (or angles) that are observed in the model and the number of bonds (or angles) that are defined in the chemical component dictionary. The Link column lists molecule types, if any, to which the group is linked. The Z score for a bond length (or angle) is the number of standard deviations the observed value is removed from the expected value. A bond length (or angle) with $|Z| > 2$ is considered an outlier worth inspection. RMSZ is the root-mean-square of all Z scores of the bond lengths (or angles).

Mol	Type	Chain	Res	Link	Bond lengths			Bond angles		
					Counts	RMSZ	$\#\|Z\| > 2$	Counts	RMSZ	$\#\|Z\| > 2$
2	FRU	A	401	-	11,12,12	1.50	2 (18%)	10,18,18	1.03	1 (10%)

In the following table, the Chirals column lists the number of chiral outliers, the number of chiral centers analysed, the number of these observed in the model and the number defined in the chemical component dictionary. Similar counts are reported in the Torsion and Rings columns. '-' means no outliers of that kind were identified.

Mol	Type	Chain	Res	Link	Chirals	Torsions	Rings
2	FRU	A	401	-	-	0/5/24/24	0/1/1/1

All (2) bond length outliers are listed below:

Mol	Chain	Res	Type	Atoms	Z	Observed(Å)	Ideal(Å)
2	A	401	FRU	O3-C3	-2.50	1.37	1.42
2	A	401	FRU	O4-C4	-2.30	1.37	1.43

All (1) bond angle outliers are listed below:

Mol	Chain	Res	Type	Atoms	Z	Observed(^{o})	Ideal(^{o})
2	A	401	FRU	C6-C5-C4	-2.83	108.22	115.05

There are no chirality outliers.

There are no torsion outliers.

There are no ring outliers.

No monomer is involved in short contacts.

5.7 Other polymers (i)

There are no such residues in this entry.

5.8 Polymer linkage issues (i)

There are no chain breaks in this entry.

6 Fit of model and data (i)

6.1 Protein, DNA and RNA chains (i)

In the following table, the column labelled '#RSRZ> 2' contains the number (and percentage) of RSRZ outliers, followed by percent RSRZ outliers for the chain as percentile scores relative to all X-ray entries and entries of similar resolution. The OWAB column contains the minimum, median, 95th percentile and maximum values of the occupancy-weighted average B-factor per residue. The column labelled 'Q< 0.9' lists the number of (and percentage) of residues with an average occupancy less than 0.9.

Mol	Chain	Analysed	<RSRZ>	#RSRZ>2			OWAB(Å^2)	Q<0.9
1	A	308/323 (95%)	0.12	15 (4%)	30	37	20, 30, 56, 121	0

All (15) RSRZ outliers are listed below:

Mol	Chain	Res	Type	RSRZ
1	A	16	ASP	6.7
1	A	214	GLN	3.6
1	A	17	SER	2.9
1	A	106	ASP	2.8
1	A	142	CYS	2.7
1	A	221	GLN	2.7
1	A	255	ALA	2.6
1	A	280	GLN	2.6
1	A	215	SER	2.5
1	A	105	LEU	2.4
1	A	216	ILE	2.4
1	A	36	GLN	2.3
1	A	256	VAL	2.2
1	A	218	GLU	2.2
1	A	238	LYS	2.1

6.2 Non-standard residues in protein, DNA, RNA chains (i)

There are no non-standard protein/DNA/RNA residues in this entry.

6.3 Carbohydrates (i)

There are no carbohydrates in this entry.

6.4 Ligands (i)

In the following table, the Atoms column lists the number of modelled atoms in the group and the number defined in the chemical component dictionary. LLDF column lists the quality of electron density of the group with respect to its neighbouring residues in protein, DNA or RNA chains. The B-factors column lists the minimum, median, 95th percentile and maximum values of B factors of atoms in the group. The column labelled 'Q< 0.9' lists the number of atoms with occupancy less than 0.9.

Mol	Type	Chain	Res	Atoms	RSCC	RSR	LLDF	B-factors($Å^2$)	Q<0.9
3	NA	A	402	1/1	0.96	0.15	1.67	33,33,33,33	0
2	FRU	A	401	12/12	0.93	0.16	-0.10	19,28,32,33	0

6.5 Other polymers (i)

There are no such residues in this entry.
